# Mouse APOBEC3 interferes with autocatalytic cleavage of murine leukemia virus Pr180gag-pol precursor and inhibits Pr65gag processing

**DOI:** 10.1371/journal.ppat.1008173

**Published:** 2019-12-12

**Authors:** Yoshiyuki Hakata, Jun Li, Takahiro Fujino, Yuki Tanaka, Rie Shimizu, Masaaki Miyazawa

**Affiliations:** 1 Department of Immunology, Kindai University Faculty of Medicine, Osaka-Sayama, Osaka, Japan; 2 Ijunkai Medical Oncology, Endoscopy Clinic, Sakai-ku, Sakai, Osaka, Japan; 3 Division of Analytical Bio-Medicine, Advanced Research Support Center (ADRES), Ehime University, Shitsukawa, Toon, Ehime, Japan; 4 Kindai University Anti-Aging Center, Higashiosaka, Osaka, Japan; University of Illinois at Chicago College of Medicine, UNITED STATES

## Abstract

Mouse APOBEC3 (mA3) inhibits murine leukemia virus (MuLV) replication by a deamination-independent mechanism in which the reverse transcription is considered the main target process. However, other steps in virus replication that can be targeted by mA3 have not been examined. We have investigated the possible effect of mA3 on MuLV protease-mediated processes and found that mA3 binds both mature viral protease and Pr180gag-pol precursor polyprotein. Using replication-competent MuLVs, we also show that mA3 inhibits the processing of Pr65 Gag precursor. Furthermore, we demonstrate that the autoprocessing of Pr180gag-pol is impeded by mA3, resulting in reduced production of mature viral protease. This reduction appears to link with the above inefficient Pr65gag processing in the presence of mA3. Two major isoforms of mA3, exon 5-containing and -lacking ones, equally exhibit this antiviral activity. Importantly, physiologically expressed levels of mA3 impedes both Pr180gag-pol autocatalysis and Pr65gag processing. This blockade is independent of the deaminase activity and requires the C-terminal region of mA3. These results suggest that the above impairment of Pr180gag-pol autoprocessing may significantly contribute to the deaminase-independent antiretroviral activity exerted by mA3.

## Author summary

Soon after the identification of the polynucleotide cytidine deaminase APOBEC3 as a host restriction factor against *vif*-deficient HIV, it was noticed that deamination-independent mechanisms are involved in the inhibition of viral replication in addition to the deaminase-dependent mechanism. We previously showed that mouse APOBEC3 (mA3) physiologically restricted mouse retrovirus replication in their natural hosts without causing significant G-to-A hypermutations. Inhibition of reverse transcription is reported to be the most plausible mechanism for the deamination-independent antiretroviral function. However, it remains unknown whether the inhibition of reverse transcription is the only way to explain the whole picture of deamination-independent antiviral activity exerted by APOBEC3. Here we show that mA3 targets the autoprocessing of Pr180gag-pol polyprotein. This activity does not require the deaminase catalytic center and mainly exerted by the C-terminal half of mA3. mA3 physically interacts with murine retroviral protease and its precursor Pr180gag-pol. mA3-induced disruption of the autocatalytic Pr180gag-pol cleavage leads to a significant reduction of mature viral protease, resulting in the inhibition of Pr65gag processing to mature Gag proteins. As the Pr180gag-pol autoprocessing is necessary for the maturation of other viral enzymes including the reverse transcriptase, its inhibition by host APOBEC3 may precede the previously described impairment of reverse transcription. Our discovery may lead to the development of novel antiretroviral drugs through the future identification of detailed molecular interfaces between retroviral Gag-Pol polyprotein and APOBEC3.

## Introduction

Apolipoprotein B mRNA editing enzyme, catalytic polypeptide-like 3 (APOBEC3) belongs to the APOBEC family of cellular proteins, which also includes activation-induced cytidine deaminase (AID), APOBEC1, APOBEC2 and APOBEC4. Humans have seven *APOBEC3* gene loci in a tandem array on chromosome 22, while only a single gene is identified in the haploid mouse genome. All human and mouse APOBEC3 members can convert cytosines in single-stranded DNA to uracils, and this enzymatic activity contributes to the editing of the genomes of several different types of viruses including retroviruses, papillomaviruses, herpesviruses and retrotransposons [1–7]. The deaminase activity of APOBEC3 relies on its conserved zinc-coordinating domain, H-X_1_-E-X_25-31_-C-X_2-4_-C (X represents a non-conserved amino acid), which is further classified into Z1, Z2 and Z3 domains based on their interspecies homologies [8, 9]. Human APOBEC3B (Z2Z1), APOBEC3D (Z2Z2), APOBEC3F (Z2Z2), APOBEC3G (Z2Z1) as well as mouse APOBEC3 (mA3: Z2Z3) are double-domain enzymes, while human APOBEC3A (Z1), APOBEC3C (Z2) and APOBEC3H (Z3) consist of a single domain. APOBEC3G is the first identified intrinsic HIV-1 restriction factor that is counteracted by the viral infectivity factor (Vif) [7]. In the absence of Vif, APOBEC3G is incorporated into newly budding HIV-1 virions *via* interactions with viral nucleocapsid (NC) and genomic RNA [10–13], and transferred into target cells of infection. In the infected host cells, APOBEC3G deaminates cytosines to uracils in the nascent minus-strand viral cDNA during reverse transcription, resulting in the production of the plus strand with high levels of G-to-A mutations [14]. These mutations may generate undesired stop codons and resultantly disturb the production of functional viral proteins. Other APOBEC3 members including APOBEC3D, APOBEC3F and the product of some *APOBEC3H* alleles, as well as mA3, work essentially in a manner similar to APOBEC3G in restricting the replication of *vif*-deficient HIV-1 [15–18]. On the other hand, there have been multiple reports indicating that APOBEC3 restricts virus replication through deamination-independent mechanisms [19–25]. A current model for the deamination-independent antiviral activity employs oligomerization of APOBEC3 on viral genomic RNA and minus-strand DNA, which may sterically block the elongation and accumulation of the products of reverse transcription [26]. It was also reported that APOBEC3 directly binds to the reverse transcriptase (RT), and then inhibits its enzymatic activity [27, 28]. However, it remains unknown whether deamination-independent antiviral action of APOBEC3 is solely attributed to reverse transcription as the target process.

Mouse APOBEC3 shows two major allelic variations among inbred strains that are linked with resistance or susceptibility to gammaretroviruses such as Friend (F-) and Moloney (M-) murine leukemia viruses (MuLVs) as well as to a betaretrovirus, mouse mammary tumor virus (MMTV) [29–33]. The polymorphisms observed between these two alleles dictate the expression levels of mA3 mRNA and its splicing patterns [34, 35]. In virus-resistant C57BL/6 (B6) mice, mA3 mRNA is more abundant than in susceptible BALB/c and A strains of mice, and expressed mainly as an exon 5-skipped form (Δ5 mA3) [31, 33, 36]. In contrast, the exon 5-containing full-length mA3 (5+ mA3) mRNA is the major isoform detected in BALB/c mice. Higher expression levels of mA3 mRNA in B6 mice correlate with the insertion of the long terminal repeat of an endogenized xenotropic MuLV within the *APOEBC3* gene locus, which is absent from the BALB/c locus [35]. We previously reported two genetic polymorphisms that determine the exon 5 inclusion during mA3 mRNA splicing, and that exon 5 has the ability to reduce the translation efficiency [34]. Of two Z domains that constitute mA3, the N-terminal Z2 domain of either 5+ or Δ5 mA3 protein is catalytically active while the C-terminal Z3 domain is responsible for the packaging of mA3 into virions. This Z domain positioning is fully reverse of that in human APOBEC3G [37]. Interestingly, we and others have reported that G-to-A hypermutations in proviruses do not necessarily accompany the suppression of F-MuLV or M-MuLV replication in the presence of mA3 [33, 38–40]. MMTV replication is also suppressed by mA3 while G-to-A mutations in the integrated proviral genome are rarely detected [23]. These imply that a deamination-independent antiviral mechanism is the dominant way through which mA3 inhibits these exogenous murine retroviruses.

Retroviruses have evolved specialized mechanisms to evade the restriction by APOBEC3 proteins of their natural hosts. HIV and SIV encode Vif that degrades host’s APOBEC3s in a species-specific manner through ubiquitin-mediated proteasome pathway [41–48]. In the case of MuLV, glycosylated Gag polyprotein that contains 88 additional amino acids at the N-terminus of Pr65gag is reported to protect the viruses from mA3 [49–51]. It has also been reported that MuLV protease cleaves 5+ mA3 protein at the portion encoded by exon 5, leading to the production of nonfunctional N- and C-terminal single-domain fragments within virions [52]. However, there is a conflicting report indicating that mA3 fragmentation by viral protease was not significantly detectable [38]. Thus, it remains obscure whether 5+ mA3 cleavage by viral protease does occur and whether it is a general strategy through which MuLVs resist host mA3. If the exon 5-encoded region is indeed cleaved by viral protease, it indicates a direct interaction between MuLV protease and mA3. However, the possible direct association of viral protease with mA3 has not been examined. More importantly, if these proteins bind with each other, mA3 may influence the protease-mediated processes of retrovirus replication in a deaminase-independent manner.

MuLV protease is initially translated as a part of Pr180gag-pol polyprotein and cleaved to the mature form of approximately 15 kDa through its autocatalytic activity [53]. This autoprocessing is thought to rely on the activity of viral protease itself embedded within Pr180gag-pol, and homodimerization of the polyprotein is a prerequisite for the embedded protease to be active [54, 55]. The order of autoprocessing among several cleavage sites within the precursor is strictly regulated for the generation of functional viral proteins [56]. Once mature MuLV protease is autoprocessed from the polyprotein, it cleaves the Gag precursor Pr65gag into the matrix (p15), p12, capsid (p30), and NC proteins. Thus, viral protease mediates both autocatalytic cleavage of Pr180gag-pol and the processing of Pr65gag.

In the present study, we first investigated the possible physical association of mA3 with MuLV protease, and then evaluated the potential inhibitory effect of mA3 on MuLV protease-mediated processes. We report here that, irrespective of the presence or absence of exon 5-encoded region, mA3 directly binds viral protease. We also show that mA3 targets the autocatalytic cleavage of Pr180gag-pol and inhibits Pr65gag processing in a deaminase-independent manner. Further, mA3 interacts with Pr180gag-pol mainly through its C-terminal half and resultantly reduces the production of mature viral protease. We also show that physiologically expressed levels of mA3 reduces the amount of mature viral protease and inhibits Pr65gag processing in MuLV virions. Our results indicate that mA3 is an intrinsic inhibitor of retroviral protease function, which is a novel mechanism that explains at least one aspect of its deamination-independent antiretroviral activity.

## Results

### Protease sequence variations among F-MuLV molecular clones

**Fig 1:**
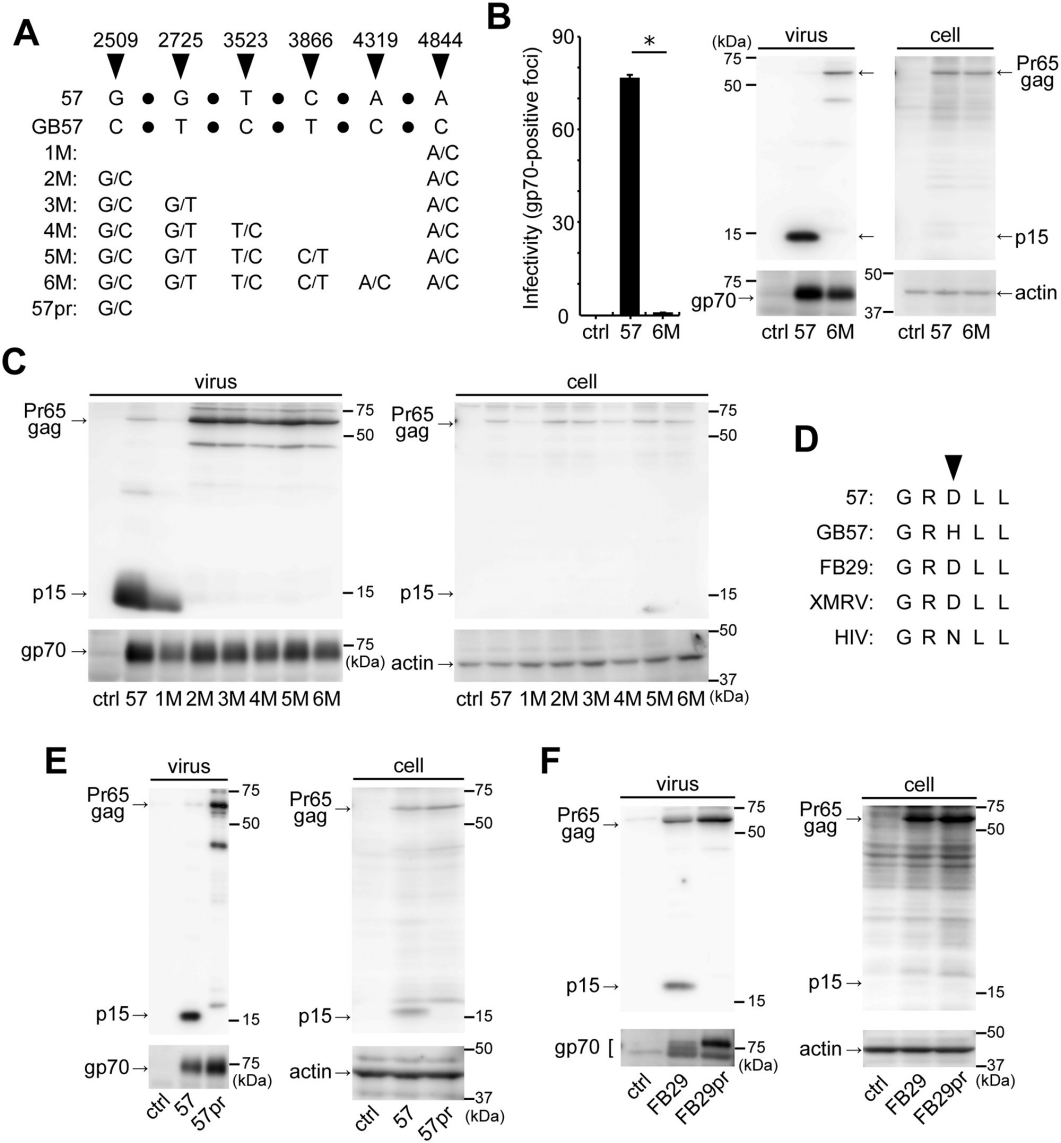
Infectious F-MuLV molecular clones and their protease-deficient mutants. (A) A diagrammatic alignment of p57(2LTR)Sp72 (57) and GB57 sequences. The latter is enrolled in GenBank (accession number X02794). Discrepancies are indicated by arrowheads and corresponding nucleotide position numbers. Sequences in between these discrepant residues are omitted and represented by filled circles. Mutants (1M to 6M and 57pr) were generated with the strain 57 sequence as the backbone with the indicated GB57-type nucleotide substitutions. The strain 57 sequence in p57(2LTR)Sp72 has been registered in DDBJ with the accession number LC229035. (B) Infectivity of strain 57 and 6M viruses. Viruses were produced from 293T cells transfected with either one of the virus-encoding plasmids. As a control (ctrl), the cells were mock-transfected with a control vacant plasmid. A portion of the culture supernatant was used for the infectivity assay in which virus infectivity was evaluated by counting the number of gp70-positive foci on *Mus dunni* cells. Results are shown as a mean with standard error of more than 3 wells. *, *P* = 0.0004 by Student’s *t*-test. Experiments were repeated three times with similar results. The remaining virus-containing supernatant was subject to ultracentrifugation to collect the viruses. The pelleted viruses and the transfected 293T cells were lysed for immunoblotting. To detect the matrix protein (p15) and its precursor, Pr65gag, the anti-p15 mAb (clone 690) was used. The envelope protein (gp70) was also detected to monitor virus production using the anti-F-MuLV gp70 mAb (clone 720). Actin is used as a loading control for the cells. (C) Detection of p15 in mutant virus particles. Viruses were produced by transient transfection and collected from the culture supernatant, and analyzed as described for panel B. The cell lysates were also analyzed as described for panel B. (D) Variations in amino acid residue at position 96 (indicated by the arrowhead) within MuLV protease. For HIV, the amino acid residue indicated by the arrowhead is located at position 88 of viral protease. FB29, GenBank accession number Z11128; XMRV, GenBank accession number NC_007815.1; HIV-1 strain NL4-3, GenBank accession number M19921. (E) A single nucleotide substitution at position 2509 that results in the D-to-H amino acid substitution at position 96 of the viral protease inactivates Pr65gag processing in F-MuLV strain 57. The mutant, 57pr, harbors the D-to-H substitution at position 96 of the protease. Viruses were produced by transient transfection and collected by ultracentrifugation. The virus and cell lysates were subjected to immunoblotting. Anti-p15 (MA) mAb clone 690, anti-F-MuLV gp70 (SU) mAb clone 720 and anti-actin Ab C-11 were used to detect p15 and Pr65gag, gp70, and cellular actin, respectively. (F) The same analyses as shown in panel E except that FB29 and its mutant (FB29pr) which harbors the D-to-H substitution at position 96 of the protease were used. A faint band discernible close to the molecular weight of Pr65gag in the control lane is a non-specific signal.

Although the cleavage of 5+ mA3 by MuLV protease may indicate direct physical interactions between these two proteins, whether this cleavage occurs remains an open question [38, 52]. To clarify this in a manner as close to natural MuLV infection as possible, we chose two replication-competent strains of F-MuLV, 57 and FB29. We have been routinely using FB29 and confirmed it to be infectious [33, 57]. Strain 57 is replication-competent and pathogenic, and its nucleotide sequence is enrolled in the GenBank (accession number X02794), which we designate GB57 (GenBank 57) hereafter [58]. We obtained a molecular clone of strain 57 from Marc Sitbon, L’institut de Génétique Moléculaire de Montpellier [59], and reanalyzed its nucleotide sequence (DDBJ accession number LC229035). We found that the *pol* region of this molecular clone has six nonsynonymous nucleotide substitutions as compared to GB57 (Fig 1A). The most upstream of the six substitutions is found within the *protease* region (at position 2509: the nucleotide numbers shown are from the first nucleotide in the 5’ LTR of GB57) and the others are located in the *reverse transcriptase* (at positions 2725, 3523, 3866, and 4319) or *integrase* (at position 4844) region. Since it is important for the following experiments to examine if these nucleotide substitutions influence viral infectivity, we first substituted all the six nucleotides in the strain 57 to the corresponding GB57 residues, generating the clone 6M (Fig 1A). We transfected 293T cells with these original and mutant molecular clones, and viruses produced in the supernatant were subjected to immunoreactive focus assays (Fig 1B). The unmodified virus strain 57 produced a large number of infectious foci while the clone 6M produced almost no detectable focus. We detected p15 in the 57 but not in 6M virion lysate, although unprocessed Pr65gag was detected in the latter lysate. Gp70 and actin were used as loading controls for virions and cell lysates, respectively, and Pr65gag was equally detected in the transfected cells. These results indicate that viral protease activity is compromised in clone 6M. We next prepared a series of mutant molecular clones, designated 1M through 5M, by successively including nucleotide substitutions from the 57 to GB57 (Fig 1A), and Pr65gag processing in these viruses was evaluated (Fig 1C). All mutants expressed Pr65gag in transfected cells in various amounts. Virions of clone 1M showed Pr65gag processing identical to that observed in strain 57 virions, while 2M, 3M, 4M and 5M virions, all commonly carrying the G/C substitution at position 2509, failed to show Pr65gag processing. These results indicate that the nucleotide substitution at position 2509 may cause the inactivation of viral protease. Nucleotide position 2509 is a part of the codon encoding the 96th amino acid in MuLV protease (Fig 1D, arrowhead). F-MuLV 57 encodes an aspartic acid while the GB57 encodes a histidine at this position. FB29 and xenotropic murine leukemia virus-related virus (XMRV) both share the aspartic acid with strain 57. The corresponding position in HIV-1 protease is the 88th amino acid which is an asparagine. To confirm that this amino acid substitution alone suffices for the inactivation of viral protease, we prepared 57pr and FB29pr molecular clones which only have the G-to-C substitution at position 2509 of strain 57 or FB29, respectively, and assessed their Pr65gag processing patterns (Fig 1E and 1F). This substitution did not disturb the expression of Pr65gag in transfected cells for both strains, and p15 was readily detected in unmutated virions of both strains 57 and FB29. The p15 signal was also visible with the lysate of 57-transfected cells in the particular experiment shown in panel E, while p15 signal is usually undetectable in cell lysates as shown in panels B, C and F. As Pr65gag cleavage is generally thought to occur during and/or after virion budding from the cell membrane, the p15 observed in cell lysates may correspond to this protein generated within virions which are under maturation process at the cell membrane. Alternatively, mature virions may be left attached to cell membranes in this particular preparation. Critically, however, p15 was not observed in the lysate of 57pr-transfected cells. p15 was also undetectable in both mutant virions while the presence of unprocessed Pr65gag was detected. These results indicate that the substitution at position 2509 alone is sufficient for the inactivation of viral protease. From these results we decided to use the strain 57, but not the virus of GB57 sequence, along with FB29 as infectious F-MuLVs in the following experiments together with 57pr and FB29pr as protease-inactivated mutant viruses in order to elucidate the possible interactions between mA3 and viral protease.

### The degree of 5+ mA3 cleavage by viral protease depends on virus strains

**Fig 2:**
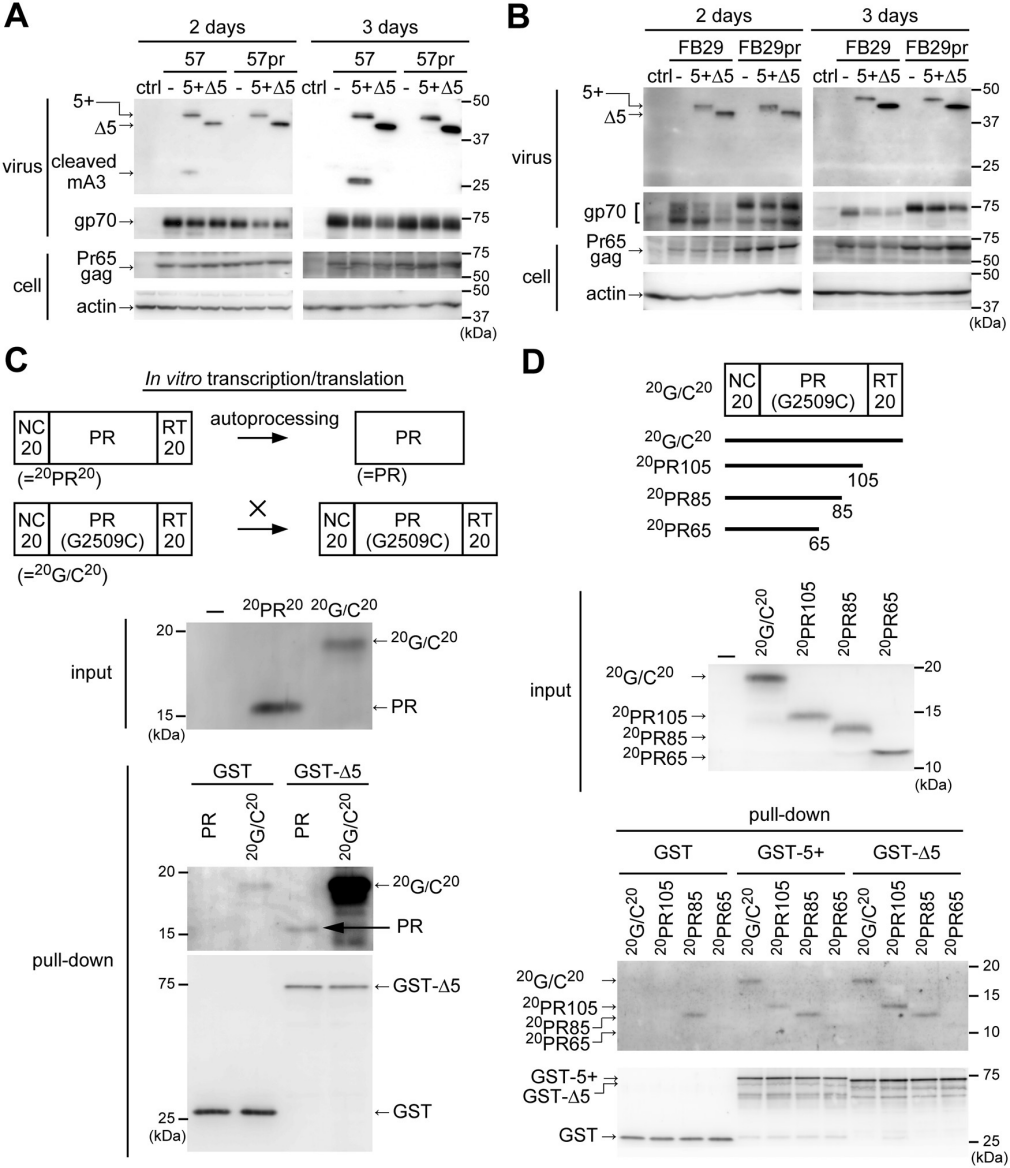
mA3 interacts with F-MuLV protease. (A) 293T cells were transfected with 6 μg of F-MuLV 57- or 57pr-encoding plasmid along with 1.5 μg of the FLAG-tagged mA3 (5+ or Δ5) expression one or the control (−) parental pCMV2-FLAG plasmid. Viruses and the cells were collected at the indicated time-points after transfection, and analyzed by immunoblotting. Anti-p15 (MA) mAb clone 690, anti-F-MuLV gp70 (SU) mAb clone 720, and anti-actin Ab C-11 were used to detect Pr65gag, gp70, and cellular actin, respectively. To detect mA3, the anti-FLAG mAb M2 was used. (B) The same experiments were performed as described for panel A except that FB29 and FB29pr were used. (C) The enzymatically active WT viral protease (PR) or the inactive G2509C mutant protein (PR (G2509C)) from strain 57 were prepared by using the *in vitro* transcription/translation system (input). Both proteins were initially expressed with twenty amino acid residues flanking each end of the protease. Since the flanking peptides of ^20^PR^20^ were autocatalytically cleaved by active PR, the processed mature viral protease migrated to produce a signal at around 15kDa. On the other hand, the mutant ^20^G/C^20^ is unable to cut the flanking peptides, resulting in the band with a molecular mass higher than that of mature viral protease. For the control (−), no DNA was added in the reaction. For the pull-down assays, GST or GST-conjugated Δ5 mA3 bound on Glutathione Sepharose 4B was mixed with the above expressed PR or ^20^G/C^20^. The associated protease in the pulled-down fraction was detected by immunoblotting using the rabbit anti-MuLV protease Ab we prepared. For the detection of GST-conjugated proteins, the anti-GST Ab was used. (D) Deletion mutants of strain 57 protease were synthesized by the *in vitro* transcription/translation system. These were mixed with GST, GST-conjugated Δ5, or GST-conjugated 5+ mA3 bound on Glutathione Sepharose 4B. The associated protease or its fragments were detected by immunoblotting as described for panel C.

Having obtained the molecular clones proficient and deficient in the protease activity, we next wished to solve the contradiction in terms if 5+ mA3 is a substrate for MuLV protease. To this end, we cotransfected 293T cells with virus-encoding and FLAG-tagged mA3-expressing plasmids. In all these experiments, we used a minimal amount of the mA3 expression plasmid to avoid the possible artificial effects induced by overexpression of mA3. Under this condition, mA3 was easily detectable in virions but was generally undetectable in cell lysates. At 2 or 3 days after transfection, virions in the supernatant were collected and mA3 cleavage evaluated by immunoblotting. The cleaved products of 5+ mA3 were readily detected in lysates of strain 57 virions with an apparently increased level of cleavage at 3 days in comparison with 2 days after transfection (Fig 2A). Pr65gag was expressed at comparable levels in the cells between samples. The cleaved mA3 products were not detected in 57pr virions even at 3 days after transfection despite their containing an amount of 5+ mA3 comparable to that in the wild-type 57 virions. These results indicate that the mA3 cleavage depends on the presence of intact viral protease. Exon 5-skipped (Δ5) mA3 appeared not to be digested. These observations are consistent with the previous report describing that mA3 was cut by viral protease at the region encoded by exon 5 [52]. In contrast, 5+ mA3 cleavage was undetectable within FB29 virions irrespective of the day of virion harvesting even though 5+ mA3 was incorporated into the virions (Fig 2B). These results indicate that the extent of mA3 cleavage varies among MuLV strains. Mechanisms underlying this strain-specific digestion of 5+ mA3 are unclear. Importantly, however, 5+ mA3 cleavage by strain 57 protease strongly suggests that these two proteins physically interact with each other.

### Viral protease interacts with mA3 irrespectively of the presence or absence of exon 5-encoded portion

To examine the presumable binding of mA3 to viral protease more directly, we performed pull-down assays in which the intact strain 57 protease and inactive counterpart carrying the G2509C substitution were prepared by *in vitro* transcription/translation reactions (Fig 2C, diagrams). We added consecutive twenty amino acids corresponding to a part of NC and RT, respectively, at the N- and C-termini of the viral protease in order to monitor the autocatalytic activity. The unmutated viral protease was designed to be synthesized with the above flanking regions (^20^PR^20^), but these flanking peptides were digested out by autoprocessing immediately after translation, yielding the mature viral protease (PR) with an apparent molecular mass of 15kDa (Fig 2C, input). On the other hand, the inactive protease mutant was not able to cut the flanking peptides, resulting in the translated product with a higher molecular weight (^20^G/C^20^) as compared to that of the active protease. GST-fused Δ5 mA3 bound to glutathione resin was incubated with either the active or mutant protease preparations that were translated *in vitro*. The active protease was detected, albeit rather faintly, in the pulled-down fraction (Fig 2C, pull-down, arrow) while the protease mutant (^20^G/C^20^) was detected as a prominent band. The active protease did not bind to GST alone. These results indicate that Δ5 mA3 binds active viral protease itself. However, it is still possible that the apparently stronger binding of ^20^G/C^20^ than that of mature protease to GST-Δ5 mA3 may be due to the interaction of Δ5 mA3 with the flanking regions rather than with the protease itself. To exclude this possibility, we generated protease deletion mutants and performed the same pull-down analysis (Fig 2D). Plasmids expressing C-terminal deletion mutants were generated by inserting a stop codon at amino acid positions 106, 86 or 66 within the protease sequence. All these deletion mutants were expressed with expected molecular masses by the *in vitro* transcription/translation reaction and detected with the rabbit anti-F-MuLV protease antibody (Ab) as they all harbored the antigenic peptide (residues 41–54)(Fig 2D, input). The deletion mutant ^20^PR105 which comprises the N-terminal flanking region and amino acid residues 1–105 of the G2509C mutant protease bound both GST-coupled 5+ and Δ5 mA3 (Fig 2D, pull-down). While we observed that ^20^PR105 bound GST-5+ mA3 with reduced affinity as compared to ^20^G/C^20^, both ^20^G/C^20^ and ^20^PR105 equally bound GST-Δ5 mA3. They did not bind GST alone. The deletion mutant ^20^PR85 bound GST alone, indicating a nonspecific association. Thus, while ^20^PR85 was detected in the samples pulled down with either GST-5+ or GST-Δ5 mA3, we could not determine if this deletion mutant binds mA3. The binding of either type of GST-coupled mA3 to ^20^PR65 mutant was hardly detectable, despite the presence of the upstream NC sequence common to the four input protease polypeptides. These results indicate that Δ5 mA3 binds viral protease itself but not the flanking regions, and the protease region downstream of the amino acid residue 65 is required for the formation of the putative binding site between mA3 and viral protease.

### Mouse APOBEC3 inhibits Pr65gag processing and perturbs Pr180gag-pol autoprocessing

**Fig 3:**
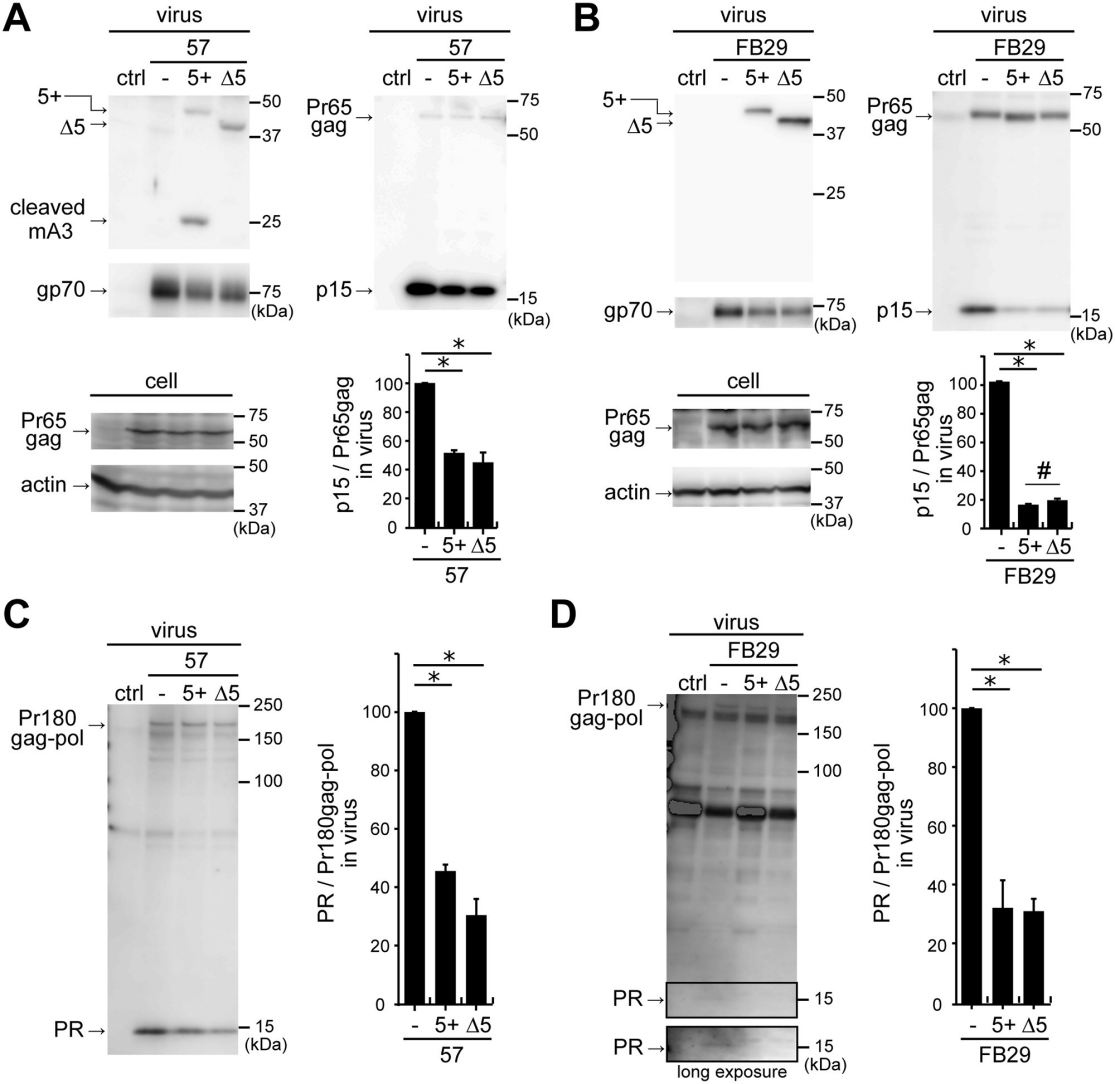
Virus-incorporated mA3 perturbs autocatalytic Pr180gag-pol cleavage and inhibits Pr65gag processing. (A) A comparison of Pr65gag processing patterns in the presence and absence of mA3. Strain 57 viruses were generated in the presence (5+ or Δ5) or absence (−) of mA3. The virions and cell lysates were analyzed by immunoblotting. Anti-p15 (MA) mAb clone 690, anti-F-MuLV gp70 (SU) mAb clone 720, anti-FLAG mAb M2, and anti-actin Ab C-11 were used to visualize p15 and Pr65gag, gp70, mA3, and cellular actin, respectively. Band intensities of p15 and Pr65gag on the same blot were measured and p15/Pr65gag ratios were calculated. The data represent means with standard errors from three independent experiments. *, *P* < 0.001 by one-way ANOVA with Tukey’s multiple comparison tests. (B) The same experiments as described for panel A except that FB29 was used. *, *P* < 0.001; #, *P* < 0.05 by one-way ANOVA with Tukey’s multiple comparison tests. (C and D) The same virus lysates were used as shown in panels A and B to detect mature viral protease and its precursor, Pr180gag-pol. The rabbit anti-MuLV protease Ab was used for immunoblotting. The image taken with a long exposure time for the detection of mature viral protease within FB29 virions was also shown. Band intensities of mature viral protease and Pr180gag-pol were measured on the same blot, and PR/Pr180gag-pol ratios were calculated. The data represent means with standard errors from three independent experiments. *, *P* < 0.001 by one-way ANOVA with Tukey’s multiple comparison tests.

As direct interaction of mA3 with F-MuLV protease was evident, we next wished to examine whether the viral protease activity was affected by mA3. Virus particles were prepared in the presence or absence of mA3, collected by ultracentrifugation, and the processing of Gag precursor Pr65gag to p15 by viral protease was evaluated. Pr65gag was expressed equally between samples in the transfected cells, and 5+ mA3 in strain 57 virions was confirmed to be cut (Fig 3A). The amount of p15 was apparently reduced when either 5+ or Δ5 mA3 was included in virions of strain 57. However when we evaluated the production of virions by detecting gp70 in the virus lysates, a reduction of gp70 in the presence of mA3 was also observed (Fig 3A, virus, gp70). This gp70 reduction suggests that mA3 may suppress virion assembly and/or budding and indicates a risk in evaluating the possible ability of mA3 to inhibit Pr65gag processing by simply measuring the amount of p15, as it reflects both virus production and the processing of Pr65gag. To overcome this complexity, we calculated ratios of band intensities corresponding to unprocessed Pr65gag and p15 in the same virus lysate (p15/Pr65gag), which enabled us to detect any changes in the Pr65gag processing efficiency in the presence of mA3. The p15/Pr65gag ratio of virions without mA3 was set at 100 (Fig 3A, bar chart). p15/Pr65gag ratios for virions containing mA3 were significantly lower than that for virions without mA3, indicating that mA3 inhibits the processing of Pr65gag to p15. Both 5+ and Δ5 mA3 were equally effective in inhibiting Pr65gag processing in strain 57 virions. Similarly, we also examined the processing of Pr65gag to p30 and found that the ratio between p30 and Pr65gag was significantly reduced in the presence of mA3 (S1 Fig). We further performed the same experiments using FB29 (Fig 3B), and obtained essentially the same results with apparently more profound inhibitory effects of both mA3 isoforms on Pr65gag processing. Interestingly, slightly reduced inhibition of Pr65gag processing to p15 was observed in the presence of Δ5 mA3 in comparison with that observed in the presence of 5+ mA3.

The production of mature viral protease by autocatalytic cleavage of Pr180gag-pol is dependent on the activity of protease portion embedded within the precursor polyprotein. Thus, we next evaluated whether this process was also targeted by mA3. For this purpose, we used the rabbit anti-F-MuLV protease Ab that is reactive to both the mature viral protease and Pr180gag-pol (see Materials and methods). Using this Ab along with the same virus lysates prepared for the experiments shown in Fig 3, panels A and B, we observed an apparent reduction of mature viral protease in F-MuLV virions in the presence of either 5+ or Δ5 mA3 (Fig 3C for 57 and 3D for FB29). The protease band detectable from FB29 virions without mA3 was faint but measurable and became discernible after a long exposure. We calculated ratios of the mature viral protease to Pr180gag-pol (PR/Pr180gag-pol) for each virus lysate in order to evaluate any possible changes in the autoprocessing efficiency in the presence of mA3 as in the case of the above evaluation of Pr65gag processing to p15. The PR/Pr180gag-pol ratio of virions without mA3 was set at 100 (Fig 3C and 3D, bar charts). PR/Pr180gag-pol ratios of virions containing 5+ or Δ5 mA3 were significantly lower than those for virions without mA3 irrespective of the virus strains, indicating that mA3 perturbs the autoprocessing of Pr180gag-pol.

To confirm that the presence of mA3 directly affects the protease function, we next examined the possible dose-dependency in the interference of Δ5 mA3 with the Pr65gag processing and Pr180gag-pol autoprocessing (S2 Fig). We used only Δ5 mA3 in these experiments since 5+ mA3 is cleaved by the viral protease, possibly making the quantitative evaluation difficult. Progressively increasing amounts of Δ5 mA3 was incorporated into 57 virions depending on the amounts of mA3-expressing plasmid, and gp70 was reduced in the presence of mA3, consistent with the results shown in Fig 3A. Pr65gag expression in the transfected cells was not apparently affected by the amounts of mA3 expression plasmid. Importantly, both p15/Pr65gag and PR/Pr180gag-pol ratios were gradually decreased when the amount of mA3 in virions increased (S2 Fig, panels B and C). These results support the notion that mA3 specifically interferes with Pr180gag-pol autoprocessing and inhibits Pr65gag processing.

To exclude the possibility that the interference of mA3 with the viral protease-mediated processes is only observed with F-MuLV, we next tested whether the production of mature protease and its enzymatic function of another MuLV, M-MuLV, were similarly targeted by mA3. M-MuLV particles were generated in the presence or absence of mA3 and virion components were evaluated by immunoblotting (S3 Fig). As observed with F-MuLV, M-MuLV incorporated mA3 when co-expressed in the producer cells, and mA3 coexpression reduced the amount of gp70. We detected a faint but discernible signal of mature protease by immunoblotting of M-MuLV particles in the absence of mA3 (S3 Fig, panel A). On the other hand, mature viral protease was undetectable in M-MuLV virions containing mA3, indicating that mA3 interferes with M-MuLV Pr180gag-pol autoprocessing similarly to the cases of F-MuLV. However, as Pr180gag-pol in M-MuLV samples was undetectable with the protease-specific Ab that worked for F-MuLV, possibly because of less efficient production of the precursor polyprotein in comparison with that in F-MuLV, we were unable to calculate PR/Pr180gag-pol ratios for M-MuLV. Nevertheless, p30/Pr65gag and p15/Pr65gag ratios were significantly reduced in the presence of either 5+ or Δ5 mA3 (S3 Fig, panels C and D), although the reduction of p30/Pr65gag ratio was slightly less pronounced in the presence of Δ5 than that observed in the presence of 5+ mA3. Thus, mA3 reduces the amount of mature protease and effectively interferes with Pr65gag processing to p15 and p30 not only in F-MuLV but also in M-MuLV.

### Physiologically expressed levels of mA3 targets the viral protease-mediated processes

**Fig 4:**
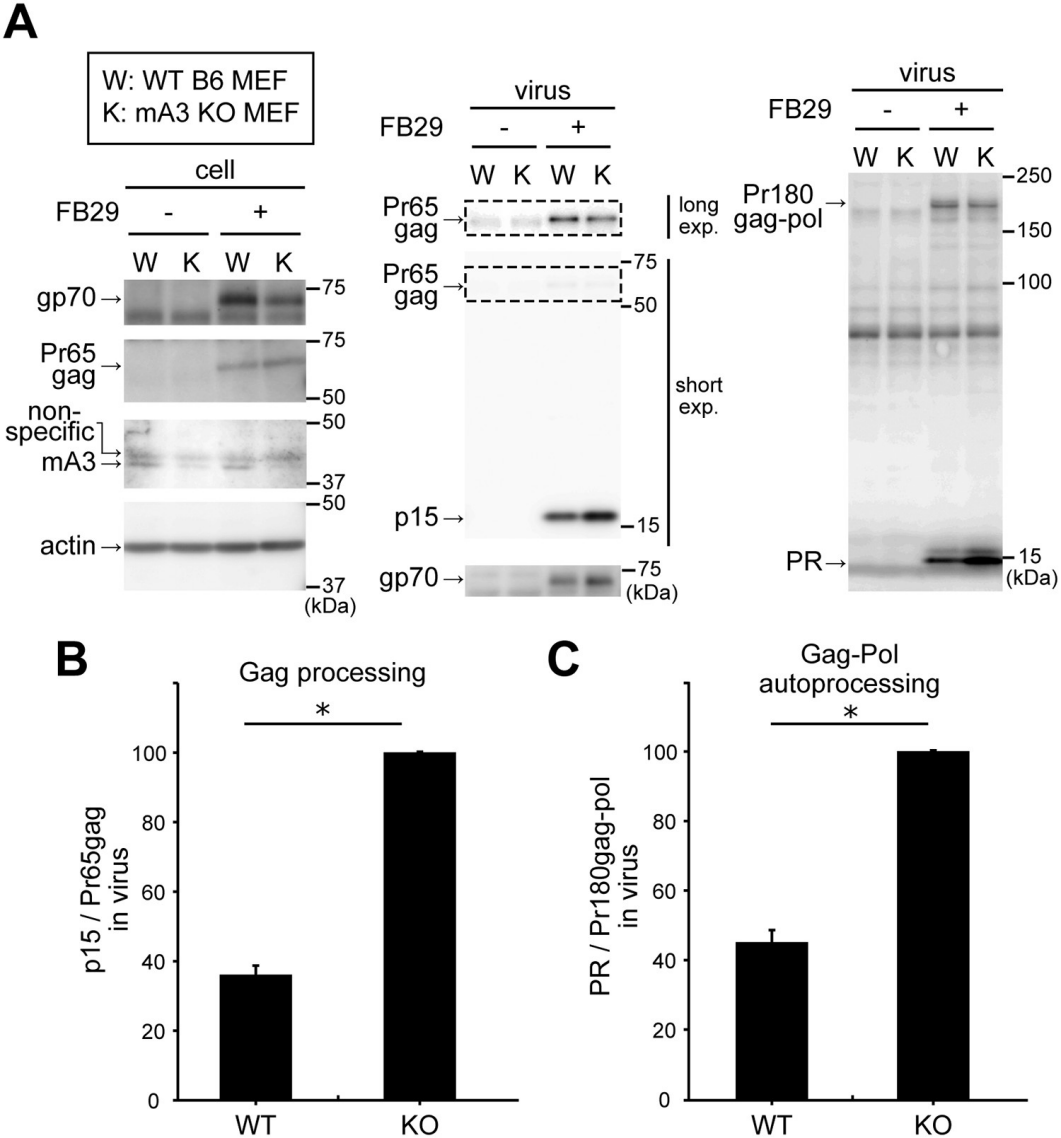
Physiologically relevant amounts of mA3 suffice for the inhibition of Pr65gag processing and perturbation of Pr180gag-pol autoprocessing. (A) MEF cells of WT (W) and mA3 KO (K) B6 mice were infected with F-MuLV FB29 (+) or mock infected (−). At a day after infection, the cells were treated with LPS. At 3 days postinfection, produced viruses and the cells were harvested and analyzed by immunoblotting. Anti-p15 (MA) mAb clone 690, anti-F-MuLV gp70 (SU) mAb clone 720, and anti-actin Ab C-11 were used to detect p15 and Pr65gag, gp70, and cellular actin, respectively. For the detection of PR and Pr180gag-pol, the rabbit anti-MuLV protease Ab was used. The anti-mA3 Ab was pre-absorbed with mA3 KO spleen extract, and used for the detection of endogenous mA3. Pr65gag signals detectable after a long exposure time are also shown. (B) Band intensities of p15 and Pr65gag on the same blot as shown in the middle part of panel A were measured, and p15/Pr65gag ratios were calculated and expressed by setting the ratio for the KO sample as 100 for each experiment. The data represent the mean with standard error from three independent experiments. *, *P* = 0.0015 by one-sample *t*-test comparing to the value 100. (C) Band intensities of mature viral protease and Pr180gag-pol on the same blot as shown in the right part of panel A were measured and PR/Pr180gag-pol ratios were calculated as above. The data represent the mean with standard error from three independent experiments. *, *P* = 0.0035 by one-sample *t*-test comparing to the value 100.

We next wished to investigate whether a physiological amount of mA3 exerts similar inhibition of the viral protease-mediated processes upon F-MuLV infection, as ratios between the amounts of viral components to mA3 could affect this function. We infected mouse embryonic fibroblasts (MEFs) prepared from mA3 knock-out (KO) or wild-type (WT) B6 mice with replication-competent FB29 virions. One day after infection, both the MEFs were treated with LPS to enhance mA3 expression [60] and continued to be cultured for 2 more days. Both the viruses generated into culture supernatant and the infected cells were analyzed. Comparable amounts of gp70 and Pr65gag were detected from the lysates of infected MEFs (Fig 4A, cell). The detection of endogenous mA3 by immunoblotting with Abs available at present is somewhat difficult since these Abs reacted nonspecifically with cellular proteins, one of which generated a signal close to the mA3 band on immunoblotting. We used the Ab which had been pre-absorbed with the spleen extract prepared from mA3 KO mice as described previously [34], allowing us to detect endogenous mA3 as a band separable from the background (Fig 4A, cell, mA3). However, the endogenous mA3 incorporated into FB29 virions produced from infected WT B6 MEF was barely detectable over the background (S4 Fig, arrow). Despite the undetectable level of endogenous mA3 incorporated into the virions, we observed apparently reduced amounts of p15 and mature viral protease in virions generated from WT MEF as compared to those in virions generated from mA3 KO MEF (Fig 4A, virus, p15 and PR). The amounts of gp70 in virions generated from WT MEF appeared to be lower than that in virions from mA3 KO MEF, which may reflect either the inefficient virus replication in WT MEF or possible mA3 function in hampering virion production as observed in the previous experiments (Fig 3A and 3B). We therefore quantified band intensities of p15, Pr65gag, mature viral protease, and Pr180gag-pol, and again calculated the ratios of p15/Pr65gag and PR/Pr180gag-pol (Fig 4B for the Gag processing and 4C for Gag-Pol autoprocessing). Both p15/Pr65gag and PR/Pr180gag-pol ratios of virions generated from WT MEF were significantly lower as compared to those of virions generated from mA3 KO MEF, indicating that physiological expression levels of mA3 inhibits the viral protease-mediated Pr65gag processing and perturbs Pr180gag-pol autoprocessing.

### Deaminase-independent perturbation of viral protease-mediated processes by mA3

**Fig 5:**
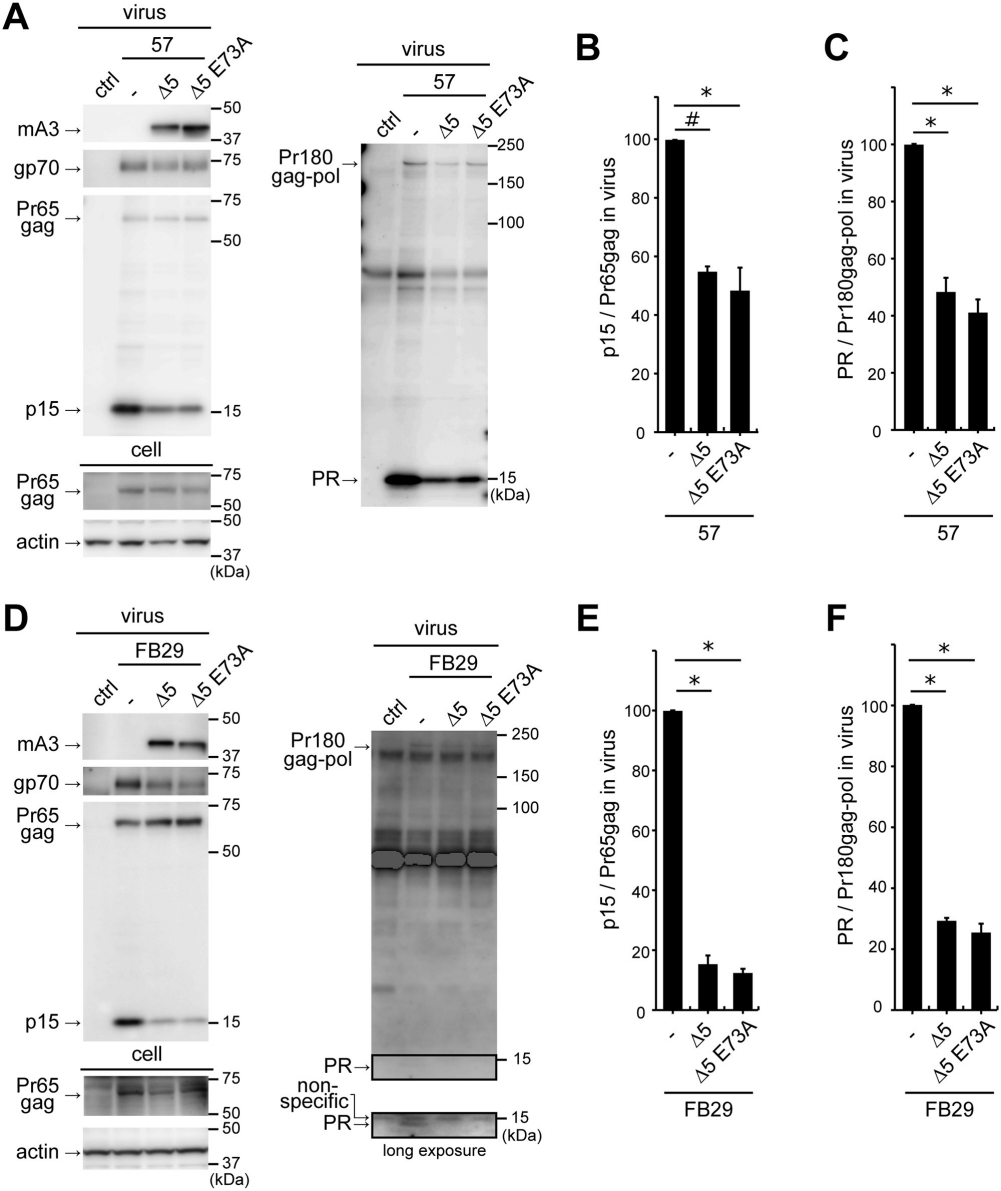
The impact of mA3 on viral protease-mediated processes was deaminase-independent. (A-C) The experiments were performed similarly to those shown in Fig 3A and 3C except by using the deaminase-inactivated E73A mutant (Δ5 E73A) along with the wild-type Δ5 mA3. The data represent means with standard errors from three independent experiments. *, *P* < 0.001; #, *P* < 0.01 by one-way ANOVA with Tukey’s multiple comparison tests. (D-F) The experiments were performed similarly to those shown in Fig 3B and 3D except by using the E73A mutant along with the wild-type Δ5 mA3. The image taken after a long exposure time for the detection of mature viral protease is also shown. *, *P* < 0.001 by one-way ANOVA with Tukey’s multiple comparison tests.

To obtain further insights into the mechanisms by which mA3 inhibits the processing of Pr65gag and perturbs the autoprocessing of Pr180gag-pol, we next tested if cytosine deaminase activity is required for this function. For this purpose, we utilized the Δ5 E73A mutant in which the glutamic acid at position 73 was substituted with an alanine [33]. This glutamic acid is located in the catalytic center, and its substitution with alanine is known to abrogate the deaminase function [37]. The Δ5 mA3 and its E73A mutant were equally encapsidated in strain 57 virions, and the amounts of p15 and mature viral protease were reduced in the presence of mA3 regardless of the amino acid residues at position 73 (Fig 5A). We calculated p15/Pr65gag (Fig 5B) and PR/Pr180gag-pol ratios (Fig 5C) and found that Δ5 E73A mutant retained as strong inhibitory effects on Pr65gag processing and Pr180gag-pol autoprocessing as Δ5 mA3 showed. We obtained similar results using FB29 with stronger inhibitory effects (Fig 5D, 5E and 5F). These results indicate that mA3 acts in a deaminase-independent manner on the perturbation of viral protease-mediated processes.

### The C-terminal half of mA3 is sufficient for targeting the viral protease-mediated processes

**Fig 6:**
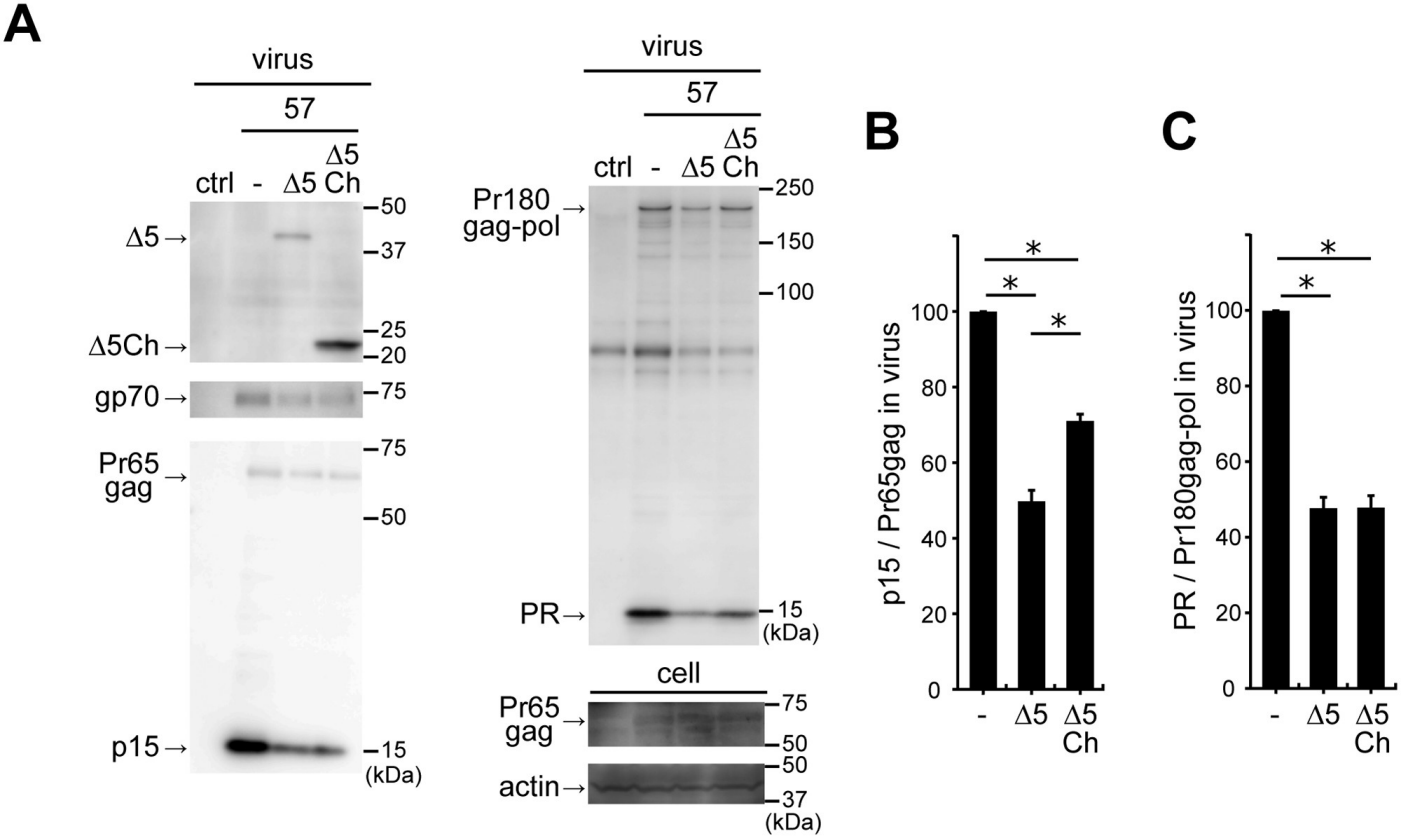
The effect of C-terminal half of mA3 on Pr65gag processing and autocatalytic Pr180gag-pol cleavage. (A-C) The experiments were performed similarly to those shown in Fig 3A and 3C except by including the C-terminal half of Δ5 mA3 (Δ5Ch). The data represent means with standard errors from three independent experiments. *, *P* < 0.001 by one-way ANOVA with Tukey’s multiple comparison tests.

We next wished to test the possible ability of the N- and C-terminal halves of mA3 in suppressing the production of mature viral protease. The C-terminal half of mA3 we generated (Δ5Ch) comprises amino acid residues from the methionine at position 200 to the end of Δ5 mA3. We prepared strain 57 virions containing Δ5 mA3 or Δ5Ch by transient transfection (Fig 6A). Both Δ5 mA3 and Δ5Ch were incorporated into virions. We did not include the N-terminal half in this experimental setting since this fragment is known not to be encapsidated into virions [37]. The amount of mature viral protease was reduced in the presence of either Δ5 or Δ5Ch. The amount of p15 was also reduced in virions containing either type of mA3. Estimation of the rates of Pr65gag processing (p15/Pr65gag, Fig 6B) and Pr180gag-pol autoprocessing (PR/Pr180gag-pol, Fig 6C) indicated that Δ5Ch showed a significant efficacy in suppressing the production of mature viral protease that was comparable to the effect of Δ5 mA3. However, although Δ5Ch significantly reduced p15, it was significantly less efficient than Δ5 in inhibiting the Pr65gag processing.

**Fig 7:**
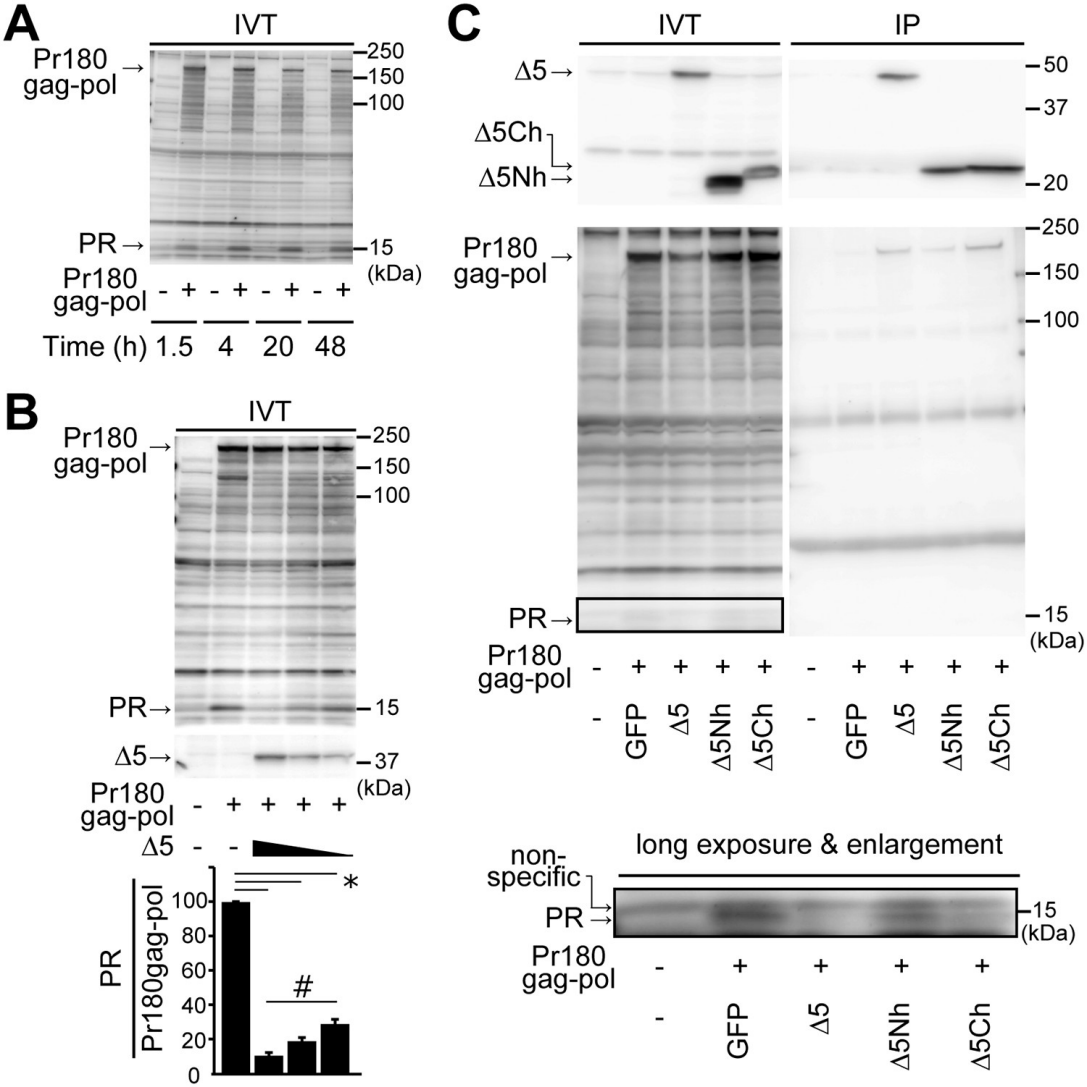
mA3 binds to Pr180gag-pol mainly through its C-terminal half and reduces mature viral protease. (A) Assays for Pr180gag-pol autoprocessing using the *in vitro* transcription and translation (IVT) system. No template DNA (−) or 1 μg of pT7CFE1-Pr180gag-pol(57) (+) was added to the IVT reaction. The reaction mixture was incubated for the indicated duration at 30°C, reaction stopped by the addition of Laemmli SDS sample buffer, and the expressed proteins analyzed by immunoblotting using the rabbit anti-MuLV protease Ab. (B) The effect of Δ5 mA3 on Pr180gag-pol autoprocessing analyzed by using the IVT system. Into the IVT reaction 0.38 μg of pT7CFE1-Pr180gag-pol(57) was added along with 0.26, 0.17 or 0.09 μg of pT7CFE1-Δ5mA3. The mixture was incubated for 20 hours at 30°C. The lysates were analyzed by immunoblotting. To detect Δ5 mA3, the anti-FLAG mAb was used as it is expressed as tagged protein. The rabbit anti-MuLV protease Ab was used to detect PR and Pr180gag-pol. The band intensities of PR and Pr180gag-pol on the same blot were measured and PR/Pr180gag-pol ratios were calculated (the bar chart). The data represent means with standard errors from three independent experiments. *, *P* < 0.001 for the three indicated comparisons; #, *P* < 0.01 by one-way ANOVA with Tukey’s multiple comparison tests. (C) Into the IVT reaction, 0.38 μg of pT7CFE1-Pr180gag-pol(57) was added (+) along with 0.2μg of pCFE-GFP control (GFP), pT7CFE1-Δ5 mA3 (Δ5), pT7CFE1-Δ5 mA3 N-half (Δ5Nh) or pT7CFE1-Δ5 mA3 C-half (Δ5Ch) expression plasmid. The mixture was incubated for 20 hours at 30°C. A portion of the reaction mixture was analyzed by immunoblotting (IVT). The image taken after a long exposure time is also shown in the bottom. Another portion of the mixture was used for the immunoprecipitation assay using the anti-FLAG Ab to precipitate FLAG-tagged mA3, and the precipitates were analyzed by immunoblotting to detect bound protease or its precursor (IP). The rabbit anti-MuLV protease Ab was used to detect PR and Pr180gag-pol. Anti-FLAG mAb M2 were used to detect flag-tagged mA3 proteins.

To evaluate the possible effect of the N-terminal half of mA3 on Pr180gag-pol autoprocessing without being affected by its lack of virion incorporation, we next employed the *in vitro* transcription/translation (IVT) system. First, we tested whether the IVT system can reproduce the autocatalytic cleavage of Pr180gag-pol and found that Pr180gag-pol was properly translated and the autoprocessing proceeded to generate mature viral protease through the time-points tested (Fig 7A). We next co-expressed Δ5 mA3 with Pr180gag-pol in the same IVT system and calculated PR/Pr180gag-pol ratios to detect any possible changes of the autoprocessing efficiency in the presence of Δ5 mA3. We found significant dose-dependent reductions in the ratio of PR and Pr180gag-pol in proportion to Δ5 mA3 expression levels (Fig 7B). These results indicate that this IVT system is applicable to assess the inhibitory effect of mA3 on the precursor polyprotein autoprocessing without the need for virion production.

Thus, we next expressed the N-terminal half of the Δ5 mA3 (Δ5Nh) that comprises the first to 198th amino acid residues as well as the full-length Δ5, Δ5Ch and GFP as a control along with Pr180gag-pol in the IVT reaction (Fig 7C, IVT). We detected mature viral protease when Δ5Nh was co-expressed (Fig 7C IVT, long exposure & enlargement). Although the reduction in the amount of mature viral protease in the presence of Δ5Nh was evident, the extent of this reduction was smaller than those observed in the presence of Δ5 mA3 or Δ5Ch. The evident reduction in the production of mature viral protease in the presence of Δ5Ch further supports the applicability of this IVT assay for the evaluation of mA3 effects on Pr180gag-pol autoprocessing, as this result is in accordance with those obtained by using virions in which Δ5Ch and Δ5 mA3 significantly reduced mature viral protease with similar extent (Fig 6C). Thus, these results indicate that, when compared to Δ5Ch, the N-terminal half has a minor effect on Pr180gag-pol autoprocessing.

Finally, we assessed the possible interaction of these mA3 fragments with Pr180gag-pol by immunoprecipitation using the same IVT lysates (Fig 7C, IP). Since all types of mA3 expressed by IVT system were FLAG-tagged, these were immunoprecipitated with the anti-FLAG Ab. None of the mA3 preparations co-precipitated detectable levels of mature viral protease probably because the amount of mature protease was too small in the IVT reaction. However, Δ5 mA3 and Δ5Ch co-precipitated Pr180gag-pol and the amounts of the precipitated precursor polyprotein were larger than that co-precipitated with Δ5Nh. These different affinities between the N- and C-terminal fragments appear to correlate with the contrasting suppressive effects on the production of mature viral protease observed in the above coexpression experiments. These results show that mA3 interacts with Pr180gag-pol mainly through its C-terminal domain and the association of mA3 with Pr180gag-pol might be directly involved in the perturbation of Pr180gag-pol autoprocessing by mA3.

## Discussion

Reverse transcription has been considered a primary target process for the deamination-independent antiretroviral action of mA3. In this study, we have demonstrated that mA3 binds MuLV Pr180gag-pol precursor polyprotein and interferes with its autocatalytic cleavage. This function of mA3 is independent of its deaminase activity and mainly carried out by its C-terminal half. Since the autoprocessing of Pr180gag-pol generates mature viral enzymatic proteins, the protease, IN and RT, our finding suggests that mA3 may initially exert its deamination-independent anti-retroviral functions at a step (the Gag-Pol precursor autoprocessing) even earlier than the previously described one (reverse transcription). The reduction of mature viral protease in the presence of mA3 likely causes the observed inefficient Pr65gag processing. Furthermore, mA3 directly binds the mature viral protease, and the protease can cleave mA3 when it contains the exon 5-encoded region. Of note, physiological levels of mA3 expression in primary embryonic fibroblasts led to the aberrant Pr180gag-pol autoprocessing that resulted in the reduction of mature viral protease and significant inhibition of Pr65gag processing in F-MuLV virions, indicating that the observed effect of mA3 on protease processing does not reflect the result of an excessive mA3 protein expression *in vitro*, but rather represents physiological significance of the presently observed novel antiretroviral activity.

Two major isoforms of mA3, Δ5 and 5+, equally disturbed Pr180gag-pol autoprocessing in virions of F-MuLV strains 57 and FB29, and inhibited subsequent Pr65gag processing to p15. It is of note that Pr65gag processing was more severely inhibited in FB29 and M-MuLV (approximately 80% reduction of p15 formation) as compared to that in strain 57 virions (approximately 50% reduction) in repeated experiments (Figs 3 and 5 and S3 Fig). On the other hand, levels of perturbation of Pr180gag-pol autoprocessing appeared not to be significantly different between the two F-MuLV strains tested (approximately 60–70% reduction of mature protease formation). The reasons for these different inhibitory effects of mA3 on Pr65gag processing between the virus stains remain unknown. However, different amounts of mA3 incorporation or intrinsically different efficiencies of Pr180gag-pol synthesis or autoprocessing may have caused a different ratio between mature protease and incorporated mA3 within virions. In fact, viral precursor polyproteins of FB29 were expressed in considerably lower amounts in the producer cells as compared to those of strain 57 in our experimental settings (S5 Fig). Although levels of perturbation of Pr180gag-pol autoprocessing in the presence of mA3 are not significantly different between the two F-MuLV strains, a relatively small amount of Pr180gag-pol in each FB29 virion may have resulted in even smaller amount of mature protease formation or more efficient abrogation of its function through direct interactions with mA3, leading to a more severe inhibition of Pr65gag processing in comparison with those occurring in strain 57 virions. In this regard, the cleavage of 5+ mA3 by viral protease within FB29 virions was apparently inefficient as compared to that in strain 57 virions, which may also result from the weaker activity of mature viral protease in FB29 virions. However, we have to separate the rate of mature protease formation through precursor autoprocessing from the specific activity of mature protease in the presence and absence of mA3 in order to make it clear that the direct interaction of mA3 with mature viral protease stoichiometrically inhibits the protease function.

If the direct binding between mA3 and mature protease indeed inhibits the viral protease function, its mechanism is of interest. The direct binding may sterically inhibit the enzymatic activity of the protease. Alternatively, the direct binding may not affect the enzymatic activity itself as 5+ mA3 was cleaved by viral protease especially in strain 57 virions. In this case, however, mA3 may sequester mature viral protease away from Pr65gag substrate in virions, resultantly perturbing the Gag processing function of the protease. The slightly reduced inhibition of Pr65gag processing to p15 in both FB29 and M-MuLV in the presence of Δ5 mA3 in comparison with that observed in the presence of 5+ mA3 carrying a definite substrate sequence may partly support this possibility. Further studies are required to separate these possible mechanisms.

As to the interaction of mature viral protease with mA3, there was indirect but suggestive evidence that viral protease cuts the exon 5-encoded region and generates the cleavage products of 5+ mA3 in MuLV virions [52]. However, another report showed that no significant cleavage of 5+ mA3 was observed [38]. Here, we showed that the 5+ mA3 cleavage was readily detected in strain 57 virions, while it was undetectable in FB29 virions in the same experimental condition. In a separate set of experiments, only faint but detectable signals of 5+ mA3 cleavage products were observed within FB29 virions (S6 Fig). These results indicate that the cleavage of 5+ mA3 by viral protease does occur but its efficiency is virus strain-dependent, which may explain the discrepancy between the two previous reports. It remains unknown, however, how critically the strain-specific cleavage of 5+ mA3 influences susceptibilities to mA3-mediated restriction among MuLV strains, especially because 5+ mA3 is less efficient in restricting F-MuLV replication than protease-resistant Δ5 mA3 [33]. Further investigation through quantitative comparison of MuLV infectivities is required to clarify this issue.

Despite the fact that Δ5 mA3 is not cleaved by viral protease, a direct interaction of Δ5 mA3 with mature viral protease was clearly observed in the pull-down assays. Δ5 mA3 bound the inactivated-protease, ^20^G/C^20^, more tightly than mature protease. This higher affinity was not due to the flanking regions included at both ends of ^20^G/C^20^ as proven by the truncation experiments. Thus, it is possible that there is a conformational difference between mature protease and ^20^G/C^20^, which might be involved in the interaction with Δ5 mA3. Alternatively, the single amino acid difference at the position 96 of viral protease may have a role in the interaction with Δ5 mA3. In fact, the C-terminal region of viral protease (amino acid residues 65–125) including the above residue appears to be involved in mA3 binding. Interestingly, 5+ mA3 showed weaker affinity especially to ^20^PR105 when compared to Δ5 mA3 in the pull-down experiment. This may indicate that binding interfaces between viral protease and mA3 may be slightly different between 5+ and Δ5 mA3.

Δ5Ch decreased the production of mature viral protease from the precursor polyprotein as efficiently as the full-length Δ5 mA3 did. The reduction in yielding mature viral protease in the presence of Δ5Nh was detectable but weaker than that caused by Δ5Ch. We also found that the Δ5Ch bound Pr180gag-pol as efficiently as Δ5 mA3 did in the immunoprecipitation assays using the IVT reaction. On the other hand, Δ5Nh showed a weaker binding property. These results indicate that the C-terminal half of Δ5 mA3 is primarily responsible for the reduction in generating mature viral protease through its interaction with Pr180gag-pol. However, it remains unidentified which embedded domain(s) within Pr180gag-pol (Gag, protease, RT or IN) is targeted by mA3 or Δ5Ch for the induction of inefficient autoprocessing. All the above domains are possible targets since Pr180gag-pol autoprocessing is known to be affected not only by the embedded protease itself but also by sequences upstream and downstream of the protease, such as the embedded RT or IN domains [61–63]. Furthermore, although we have shown here that mA3 directly binds to MuLV protease itself, we have not tested if mA3 binds the same protease sequence embedded within the precursor polyprotein. It is also possible that multiple sites within Pr180gag-pol may be involved in the binding of mA3 or Δ5Ch.

Two monomers of MuLV Pr180gag-pol form a homodimer to initiate the autocatalytic cleavage [64, 65]. The order of cleavages within the homodimer of HIV-1 Pr160gag-pol has been reported [54, 56]. When HIV-1 Pr160gag-pol containing a protease-inactivating mutation was mixed with the active recombinant viral protease *in trans*, the first site to be cleaved was located between the spacer peptide 1 and NC. We used the *in vitro* transcription and translation system to generate F-MuLV Pr180gag-pol and assessed its autoprocessing in the presence or absence of mA3. We detected a few distinctive bands that were immunoreactive to the anti-MuLV protease Ab, including those of molecular masses around 180kDa, 130kDa and 15kDa (Fig 7B). Considering the known molecular masses of the MuLV *gag-pol* gene products, the immunoreactive 180kDa and 15kDa products unarguably correspond to Pr180gag-pol and mature viral protease, respectively. As to the 130 kDa putative intermediate, based on its molecular mass, viral protease antigenicity, and the above information on HIV-1 Gag-Pol autoprocessing, we assume this to represent the NC-Pol intermediate that carries the domains from NC to IN. Alternatively, it is also possible that this putative intermediate may correspond to Gag-RT which contains the entire Gag, protease and RT domains according to its size. In any case, this putative intermediate would represent an early product of Pr180gag-pol autoprocessing. Of note, the amount of this intermediate gradually decreased along with mature viral protease as Δ5 mA3 increased, indicating that mA3 may target the step of Pr180gag-pol processing that yields the protease-harboring 130 kDa intermediate. It is possible that mA3 interferes with the formation of Pr180gag-pol homodimer by interacting with the monomer and thus inhibits the autoprocessing, resulting in the reduced production of the 130kDa intermediate and mature viral protease. Alternatively, mA3 interaction with Pr180gag-pol monomer, its homodimer, or intermediates of proteolytic processing including the above 130kDa putative intermediate may lead to structural changes of the precursor or intermediates, causing aberrant cleavage of these molecules by the embedded protease and resultant reduction of mature viral protease.

As to Pr180gag-pol autoprocessing in M-MuLV virions, we have to evaluate in more detail whether the observed reduction of mature protease is indeed due to the aberrant Pr180gag-pol autoprocessing induced by mA3, since it remains possible that the apparent reduction of mature protease in the M-MuLV lysate might have been caused mainly by inefficient virus production in the presence of mA3. However, the overt inhibition of Pr65gag processing evidenced by the significant reduction of p15/Pr65gag and p30/Pr65gag ratios in the presence of mA3 may support the notion that mA3 interferes with the precursor polyprotein autoprocessing and resultantly reduces mature protease in M-MuLV as demonstrated for F-MuLV, since the reduction of virus production *per se* is unlikely to influence the efficiency of Pr65gag processing within virions.

We and others have demonstrated that mA3 restricts exogenous MuLV and MMTV without significantly increasing levels of G-to-A hypermutation in the proviruses, indicating that a deamination-independent way is the dominant mechanism for mA3 antiviral activity on murine retroviruses [23, 33, 38–40]. In this study, aberrant Pr180gag-pol autoprocessing induced by mA3 was proven to be deaminase-independent. Among the known deaminaton-independent actions of mA3 including the previously reported inhibition of reverse transcription, we cannot determine at present to what extent the aberrant Pr180gag-pol precursor autoprocessing plays a dominant role in inhibiting F-MuLV replication, as it is inherently difficult to separate this mA3 effect from the other mechanisms of action. Since the autoprocessing is crucial to generate mature RT and IN besides viral protease, its perturbation by mA3 may conceivably lead to impaired RT functions. It is of interest to confirm that the aberrant precursor autoprocessing in the presence of mA3 leads to the reduction of other viral enzymes such as RT and IN. If this is the case, the reduction of catalytically active enzymatic components may at least partially underlie the previously described restriction mechanism that suppresses MuLV replication at or before the reverse transcription in a deaminase-independent manner. Gag-Pol precursor autoprocessing is an indispensable step for retroviruses to produce all viral enzymes and thus to replicate in host cells. Understanding how mA3 perturbs the autoprocessing process may lead us to design a new type of antiretroviral drugs.

## Materials and methods

### Ethics statements

The studies utilizing mice were carried out in strict accordance with the Act on Welfare and Management of Animals of the Government of Japan and the Regulations for the Care and Use of Laboratory Animals of Kindai University. The research protocol was approved by the institutional Animal Experimentation Committee of Kindai University Faculty of Medicine (Permit Number: KAME-26-031).

### Mice

C57BL/6NCrSlc mice were purchased from Japan SLC, Inc., Hamamatsu, Japan. The mA3 KO mouse strain used in this study has been described [33, 34, 66]. All mice were housed and bred in the Experimental Animal Facilities at Kindai University Faculty of Medicine under specific pathogen-free conditions.

### Viruses

The plasmids, p57(2LTR)Sp72 and pFB29B, harboring molecular clones of F-MuLV strain 57 and FB29, respectively, and p8.2B harboring a molecular clone of M-MuLV were kindly provided by Marc Sitbon, IGMM, France [58, 59, 67, 68]. To produce infectious virus of strain 57, viral DNA was first excised out with *Hpa*I and *Xho*I from the p57(2LTR)Sp72, and purified with phenol/chloroform followed by ethanol precipitation. The purified DNA was transfected to 293T cells as described below. pFB29B was cut with *Hind*III and purified as above before transfection. Similarly, p8.2B was cut with *Hind*III, *Eco*RI and *Sse*8387 and purified with Wizard SV Gel and PCR Clean-up System (Promega). The excised viral DNA was ligated with DNA Ligation Kit ver. 2.1 (TaKaRa) and purified with phenol/chloroform followed by ethanol precipitation before transfection. The G2509C mutation within the viral genome was introduced to p57(2LTR)Sp72 and pFB29B by using QuickChange Site-Directed Mutagenesis Kit (Stratagene) according to the manufacturer’s protocol. The oligodeoxynucleotides used for the mutagenesis were 5’-CTCTGCTAGGAAGACATTTGCTGACTAAAC-3’ and its complementary DNA. The resultant plasmids were designated p57(2LTR)Sp72(G2509C) and pFB29B(G2509C), and the viruses generated by transfection of these plasmids were designated 57pr or FB29pr, respectively. Other mutant viruses were also generated by site-directed mutagenesis as follows: The oligodeoxynucleotides and templates used in the mutagenesis are 5’-GAGCTACAGTCCTTCTTACATGCTGAACCG-3’, its complementary DNA and p57(2LTR)Sp72 as the template for the construction of the mutant virus 1M; 5’-CTCTGCTAGGAAGACATTTGCTGACTAAAC-3’, its complementary DNA and 1M as the template to generate 2M; 5’-GCATGGGACTGGCCTTTCGCCAAGCTCCTC-3’, its complementary DNA and 2M as the template to generate 3M; 5’-TAGGGACGGCAGGCCTCTGTCGCCTCTGGA-3’ and its complementary DNA and 3M as the template to generate 4M; 5’-TCTGACCAAAGACGTTGGCAAACTCACCAT-3’ and its complementary DNA and 4M as the template to generate 5M; 5’-GCCTTAAAAATGGCAGCAGGTAAGAAGCTG-3’, its complementary DNA and 5M as the template to generate 6M. All mutant virus sequences were confirmed by nucleotide sequence analysis. Replication-competent FB29 was prepared from the culture supernatant of *Mus dunni* cells persistently infected with this strain of F-MuLV as described previously [69]. The titer of FB29 stock was generally around 8 x 10^6^ fluorescent focus-forming units (ffu)/ml.

### Expression plasmids

FLAG-tagged 5+, Δ5 and Δ5 E73A mA3 expression plasmids have been described [33, 34]. The truncated cDNA encoding the C-terminal half of Δ5 mA3 was prepared by PCR using primers 5’-TTGAATTCGATGGACCCGCTAAGTGAAGAGGAATTT-3’ and 5’-GGGTCGACTCAAGACATCGGGGGTCCAAGCTGTAGGTTTCC-3’ along with pFLAG-CMV2-mA3^b^Δ5 [33] as the template. The PCR products were inserted into pFLAG-CMV2 (Sigma) at the *Eco*RI/*Sal*I cleavage site. The full-length, N-terminal half, or C-terminal half of Δ5 mA3 cDNA were amplified by PCR using primers 5’-CCCCCATATGGACTACAAAGACGATGACGACAAGATGGGACCATTCTGTCTGGGATGC-3’ (F1) and 5’-GGCTCGAGTCAAGACATCGGGGGTCCAAGCTG-3’ (R1), F1 and 5’-GGCTCGAGTCACAGAATCTCCTGAAGCTTAGAATC-3’, or 5’-CCCCCATATGGACTACAAAGACGATGACGACAAGATGGACCCGCTAAGTGAAGAGGAA-3’ and R1, respectively. As the template, pFLAG-CMV2-mA3^b^Δ5 was used for the above PCR. The PCR products were inserted into pT7CFE1-CHis (Thermo Fisher) at the *Nde*I/*Xho*I cleavage site, yielding expression plasmids pT7CFE1-Δ5 mA3, pT7CFE1-Δ5 mA3 N-half and pT7CFE1-Δ5 mA3 C-half. Gag-Pol cDNA of the strain 57 was amplified by using primers 5’-TTGCGGCCGCAATGGGCCAGACTGTTACCACCCCC-3’ and 5’-TTCTCGAGTTAGGAGGTCCCGCGGGTCAATCTTAT-3’ and p57(2LTR)Sp72 as the template. The product was inserted at the *Not*I/*Xho*I cleavage site of pT7CFE1-CHis, yielding pT7CFE1-Pr180gag-pol(57). The F-MuLV *pol* gene segment encoding the protease has an internal stop codon at its 5^th^ amino acid position, which is translated as a glutamine through the read-through mechanism, resulting in the intact protease expression at a low frequency. To prepare viral protease using the *in vitro* transcription/translation system, we initially substituted the above stop codon within p57(2LTR)Sp72 and 57(2LTR)Sp72(G2509C) with CAG by mutagenesis. The oligodeoxynucleotides used for this reaction were 5’-TGACCTTAGACGATCAGGGAGGTCAGGGT-3’ and its complementary DNA. The resultant plasmids were designated p57(2LTR)Sp72(CAG) and p57(2LTR)Sp72(G2509C)(CAG), respectively, and used later as the template for PCR to generate DNA to be transcribed and translated *in vitro*.

### Preparation of mouse embryonic fibroblast (MEF) cells and cell culture

MEF cells were prepared from embryos of B6 and mA3 KO mice in our laboratory following the standard procedure [70]. In brief, minced mouse embryos were washed and treated with the trypsin buffer (0.1% trypsin, 1 mM EDTA, and 1 g/l glucose in Dulbecco’s PBS). After trypsinization, one tenth volume of DMEM (Life Technologies) supplemented with 10% fetal bovine serum (FBS, Sigma) was added to the cell suspension. The MEF cells were washed once with fresh DMEM with 10% FBS, and stocked in CELLBANKER 1 (Nippon Zenyaku Kogyo) until use. 293T (ATCC CRL-3216) human embryonic kidney cells were purchased from ATCC. *Mus terricolor* skin fibroblast cell line that is commonly called *Mus dunni* cell line [71] was kindly provided by Dr. John L. Portis, NIH, NIAID, Rocky Mountain Laboratories, Hamilton, MT. *Mus dunni* cells persistently infected with FB29 have been described [69]. These cells were cultured in DMEM containing 10%FBS in a CO_2_ incubator at 37°C. LPS (O26:B6) was obtained from Sigma and was used at 1 μg/ml.

### Virus production by transient transfection

All viral DNAs to be transfected were purified with ethanol precipitation as described above. At one day prior to transfection, 5 x 10^5^ 293T cells were seeded in a 100-mm Φ plate. The cells were transfected with 6 μg of the purified viral DNA with or without 1.5 μg of an APOBEC3 expression plasmid by using Lipofectamine 2000 or 3000 (Invitrogen), or by using FuGENE HD Transfection Reagent (Progema) for the experiment shown in S6 Fig. For all transfection experiments, the amount of total input DNA was kept constant between samples by the addition of a control empty plasmid. When a six-well plate was used, 293T cells seeded at 10^5^ per well were transfected with 1 μg of viral DNA. Three days after transfection, the culture supernatant was briefly centrifuged at 2,000 rpm for 5 min to remove cell debris and was filtered through 0.45μm pore-sized membrane (Millipore). Viruses in the filtered supernatant were concentrated by ultracentrifugation through 20% sucrose in PBS at 27,000 rpm for 2 h (SCP70H, HITACHI).

### Immunoblotting

After ultracentrifugation, pelleted viruses were lysed in the Triton lysis buffer (50 mM Tris pH 8.0, 150 mM NaCl, 0.5% Triton X-100, and 2 mM EDTA) supplemented with complete ULTRA protease inhibitor cocktail (Roche). After 10 min of incubation at room temperature, the virus lysate was mixed with Laemmli SDS sample buffer. The transfected cells were washed with PBS and lysed in the Triton lysis buffer supplemented with complete ULTRA protease inhibitor cocktail for 30 min at 4°C. The cell lysate was collected and cleared by centrifugation at 10,000 g for 10 min at 4°C. The cleared cell lysate was mixed with Laemmli SDS sample buffer. These cell and virus lysates were boiled for 5 min, and then subjected to SDS-PAGE. The separated proteins in the gel were transferred to Immobilon-P PVDF membrane (Millipore) at constant 30 V overnight. Blotted membranes were probed with a primary Ab for 2 hours at room temperature followed by washing and incubation with horseradish peroxidase-conjugated secondary Ab (Invitrogen) for 1 hour. After washing, Luminata Forte Western HRP substrate (Millipore) was added, and the protein band signals were detected under the ImageQuant LAS 4000 system (GE Healthcare). Band intensities were quantified with ImageQuant TL software (GE Healthcare).

### Antibodies and anti-F-MuLV protease antiserum

Monoclonal Ab (mAb) specific for MuLV Gag and Env proteins such as the anti-p15 (MA) clone 690, anti-p30 (CA) clone R18-7, and anti-gp70 (SU) clone 720 were prepared from culture supernatant of the corresponding hybridomas [72–75]. Affinity-purified goat anti-Rauscher gp70 Ab was originally purchased from Quality Biotech, Inc. and was kindly provided by Dr. Takase-Yoden, Soka University, Tokyo. Anti-FLAG mAb M2 was obtained from Sigma. Anti-GST Ab was obtained from GE Healthcare. Anti-actin Ab (C-11) was purchased from Santa Cruz Biotechnology. For the generation of anti-MuLV protease antiserum, we utilized a 14-mer peptide corresponding to amino acid positions 41–54 of the F-MuLV protease (TQNPGPLSDKSAWV) as the immunogen. A rabbit was immunized four times with the synthesized peptide conjugated to keyhole limpet hemocyanin and the serum was collected (Eurofins Genomics). IgG was affinity purified from the above antiserum with rProtein A-conjugated Sepharose Fast Flow (GE Healthcare). After generation, this anti-MuLV protease Ab was validated by using 57, FB29, 57pr and FB29pr viruses. We detected protein bands with apparent molecular weight of around 15kDa and 180kDa in the lysates of wild-type 57 and FB29 that were immunoreactive to the anti-MuLV protease Ab. However, in the same experimental condition, the smaller protein of around 15kDa became undetectable while the larger protein of around 180kDa remained detectable with increased intensities when protease-mutated 57pr or FB29pr was used (S7 Fig). Further, although their apparent molecular masses are close to each other, the band of p15 MA detectable with mAb 690 was separable from the band of a slightly smaller molecular mass reactive to the anti-MuLV protease Ab (see Figs 3 and 4, for example). Thus, based on the agreement of their apparent molecular masses with previous reports, reactivity of the peptide-elicited Ab, and the disappearance of the protein band of smaller molecular mass in immunoblotting in the absence of protease activity, we concluded that the bands of larger and smaller molecular mass immunoreactive to the Ab correspond to Pr180gag-pol and mature protease, respectively. To detect endogenous mA3, a pre-absorbed anti-mA3 Ab was prepared and used as previously described [34].

### Pull-down assays

GST-conjugated mA3 protein was prepared with the wheat germ cell-free protein synthesis system by following the manufacturer’s protocol (CellFree Sciences). Viral protease and its mutants were generated with an *in vitro* transcription/translation system using TNT T7 Quick for PCR DNA (Promega). The DNA templates to be translated to ^20^PR^20^ or ^20^G/C^20^ protein in this system were prepared by PCR using p57(2LTR)Sp72(CAG) or p57(2LTR)Sp72(G2509C)(CAG) as the template and the following primers: 5’-GGATCCTAATACGACTCACTATAGGGAACAGCTGGGATGAGAGATTGCCCCAAGAAGCC-3’ (F2) and 5’-CTATAGAGGCACATCTGGCCCTTTTGAGG-3’. To make the DNA template for ^20^PR105, p57(2LTR)Sp72(G2509C)(CAG) and the primers F2 and 5’-CTAAATTTGGGCTTTTAGTTTAGTCAGCAA-3’ were used. For ^20^PR85 or ^20^PR65, p57(2LTR)Sp72(CAG) and each primer set F2 and 5’-CTATACATGGAGGAAAGAGTGGGTGACCTT-3’ (for ^20^PR85) or F2 and 5’-CTACCAGCGATACCGCTTTCCTCCAGTAGC-3’ (for ^20^PR65) were used, respectively. Sixty μg of GST-conjugated mA3 bound on Glutathione Sepharose resin (GE Healthcare) was mixed with the *in vitro* transcribed/translated viral protease for 3 hours at 4°C with gentle agitation. The resin was washed with PBS containing 0.05% Tween-20 and protein complexes on the resin were dissolved in Laemmli SDS sample buffer. The resultant pulled-down proteins were analyzed by immunoblotting.

### Infectivity assays

293T cells (1x10^5^) were transfected with 2 μg of the purified virus DNA. Two days after transfection, the culture supernatant was collected and cleared by centrifugation at 1,200rom for 10 min. A portion of the cleared supernatant was treated with DNase I (TaKaRa) at 37°C for 60 min. An equal volume of the supernatant containing either 57 or 6M virus was added in the presence of 6 μg/ml Polybrene (Sigma) to a monolayer of *Mus dunni* cells seeded one day prior to infection at 1x10^4^ in a well of 24-well plates. Two days postinfection, the cells were fixed with methanol for 15 min and blocked with PBS containing 10% skim milk. After washing with PBS, the cells were incubated with anti-F-MuLV gp70(SU) mAb 720 for 2 hours at room temperature. The biotin-conjugated anti-mouse IgG was used as a secondary Ab, followed by incubation with ABC complex for 15 min (Vector Laboratories). After washing with PBS, DAB substrate was added and incubated until brown foci of infected cells were detected as previously described [75, 76]. The viruses in the remaining supernatant and the cells were harvested, and used for immunoblotting as described above to confirm that comparable levels of 57 and 6M viruses were generated in both supernatant and cell fractions.

### Infection of MEF cells with F-MuLV FB29

MEF cells (1x10^5^) were infected with replication-competent FB29 in the presence of 6 μg/ml Polybrene at an MOI of 2. Two hours postinfection, the cells were extensively washed with PBS and fed with fresh medium. One day after infection, LPS was added to the cell culture at final concentration of 1 μg/ml. Three days post infection, the culture supernatant was harvested, cleared by brief centrifugation, and then filtrated through a 0.45 μm pore-sized membrane. The filtered supernatant was loaded onto 20% sucrose in PBS and ultracentrifugation was performed at 27,000 rpm for 2 h (SCP70H, HITACHI). The virus pellets and the cells were subjected to immunoblotting as described above.

### Detection of Pr180gag-pol autoprocessing *in vitro*

Pr180gag-pol was generated with *in vitro* transcription and translation (IVT) system using 1-Step Human Coupled IVT Kit (Thermo Fisher Scientific) by following the manufacturer’s protocol. Briefly, pT7CFE1-Pr180gag-pol(57) was added to the IVT reaction mixture. When mA3 was coexpressed, pT7CFE1-Δ5 mA3, pT7CFE1-Δ5 mA3 N-half, pT7CFE1-Δ5 mA3 C-half or control pCFE-GFP was included in the reaction. The IVT reaction mixture (25μl of total volume) was incubated for the indicated duration or 20 hours unless described at 30°C. The reaction was stopped by the addition of Laemmli SDS sample buffer and the mixture was subjected to immunoblotting. For immunoprecipitation assays, 1μl of the IVT reaction mixture was diluted after translation in 1000 μl of the IP buffer (50 mM Tris pH 7.6, 150 mM NaCl, 1% IGEPAL CA-630, and 2 mM EDTA) supplemented with complete ULTRA protease inhibitor cocktail. The diluent was mixed with 40μl of the anti-FLAG mAb conjugated to agarose (Sigma) for 2 hours at 4°C with gentle agitation. After washing the agarose resin, Laemmli SDS sample buffer was added for SDS-PAGE and immunoblotting.

### Statistical analyses

Data analyses were performed by using GraphPad Prism 5 (GraphPad Software, Inc.) with an appropriate method and post-hoc test for multiple comparisons where required. The method of analysis used and obtained *P* values are shown in each figure legend.

## Supporting information

S1 Fig Virus-incorporated mA3 inhibits Pr65gag processing to p30. The virion lysates used for the experiment shown in Fig 3A were reanalyzed by immunoblotting using the anti-p30 (CA) mAb R18-7. The band intensities of p30 and Pr65gag on the same blot were measured, and p30/Pr65gag ratios were calculated (the bar chart). The data represent means with standard errors from three independent experiments. *, *P* < 0.001 by one-way ANOVA with Tukey’s multiple comparison tests. The results were consistent with those obtained with the anti-p15 mAb (Fig 3A).
(TIF)

S2 Fig mA3 interferes with Pr180gag-pol autoprocessing and inhibits Pr65gag processing in a dose-dependent manner. (A-C) The experiments were performed similarly to those shown in Fig 3A and 3C except by using varying amounts (0 (−), 0.3, 1, and 3 μg from left to right in each panel) of the Δ5 mA3-expressing plasmid added for transfection. The amount of total input DNA was kept constant between samples by the addition of the empty parental plasmid. The data represent means with standard errors from three independent experiments. *, *P* < 0.001; #, *P* < 0.01; §, *P* < 0.05 by one-way ANOVA with Tukey’s multiple comparison tests.
(TIF)

S3 Fig Pr65gag processing of Moloney MuLV is inhibited by mA3. (A-D). The experiments were performed similarly to those shown in Figs 3A and 3C and S1 Fig except that Moloney MuLV was used. The goat anti-Rauscher gp70 Ab was used for the detection of M-MuLV gp70. The data represent means with standard errors from three independent experiments. *, *P* < 0.001; #, *P* < 0.05 by one-way ANOVA with Tukey’s multiple comparison tests.
(TIF)

S4 Fig B6 MEF-derived endogenous mA3 in F-MuLV virions was barely detectable. Virus lysates prepared and evaluated as shown in Fig 4A, right panel, were used to detect mA3 in FB29 virions with the pre-absorbed anti-mA3 Ab. A band of very low intensity possibly indicating the presence of WT MEF-derived mA3 was detected, but was hardly distinguishable from the background (arrow).
(TIF)

S5 Fig When compared side-by-side FB29-producing cells expressed much lower amounts of Pr65gag than strain 57-producing cells did. 293T cells were transfected with 6 μg of viral DNA or the control vacant plasmid (ctrl). The cells were harvested at 3 days after transfection, and analyzed by immunoblotting. Anti-p15 (MA) mAb 690 and anti-actin Ab C-11 were used to detect Pr65gag and cellular actin, respectively.
(TIF)

S6 Fig 5+ mA3 cleavage in FB29 virions was detectable in a separate experimental condition. The experiment was performed similarly to that shown in Fig 2B (3 days) except by using FuGENE HD Transfection Reagent instead of Lipofectamine 3000. The virus lysates were collected at 3 days after transfection, and analyzed by immunoblotting. Anti-gp70 (SU) mAb 720 and anti-FLAG Ab M2 were used to detect gp70 and FLAG-tagged mA3 and its cleavage products, respectively. The image taken after a long exposure time for the demonstration of mA3 cleavage product is also shown in the bottom.
(TIF)

S7 Fig Validation of the rabbit anti-MuLV protease Ab. (A) The viruses were prepared as shown in Fig 2A, and analyzed by immunoblotting. Anti-gp70 (SU) mAb 720 and IgG purified from the anti-MuLV protease antiserum were used. (B) The same experiments were performed as described for panel (A) except that FB29 and the protease mutant FB29pr were used.
(TIF)
