# Supplementary figures and images for: Mouse APOBEC3 interferes with autocatalytic cleavage of murine leukemia virus Pr180gag-pol precursor and inhibits Pr65gag processing
Source: PLoS Pathog. 2019 Dec 12;15(12):e1008173. doi: 10.1371/journal.ppat.1008173 (PMC6907756; doi:10.1371/journal.ppat.1008173)

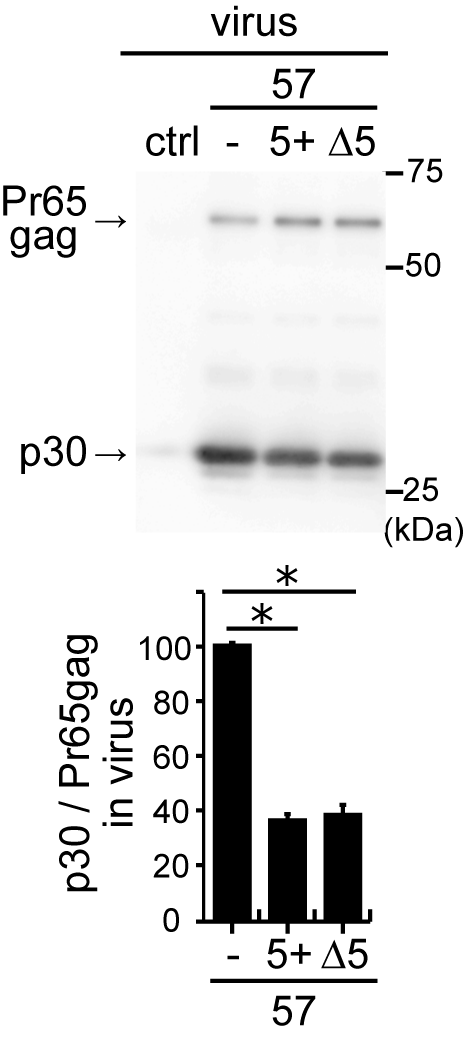

Supplement: S1 Fig — The virion lysates used for the experiment shown in Fig 3A were reanalyzed by immunoblotting using the anti-p30 (CA) mAb R18-7. The band intensities of p30 and Pr65gag on the same blot were measured, and p30/Pr65gag ratios were calculated (the bar chart). The data represent means with standard errors from three independent experiments. *, P < 0.001 by one-way ANOVA with Tukey’s multiple comparison tests. The results were consistent with those obtained with the anti-p15 mAb (Fig 3A). (TIF) [file ppat.1008173.s001.tif]

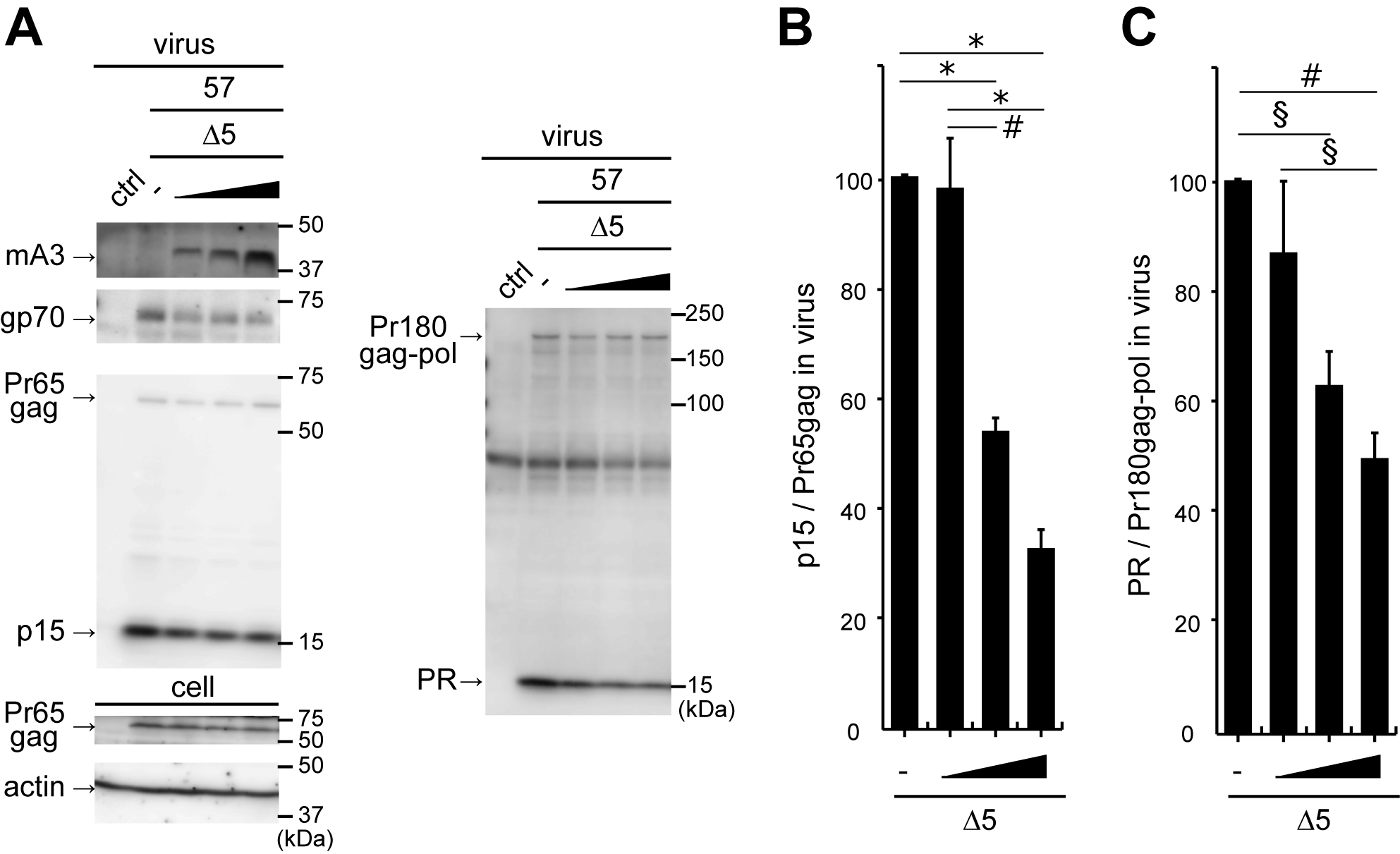

Supplement: S2 Fig — (A-C) The experiments were performed similarly to those shown in Fig 3A and 3C except by using varying amounts (0 (−), 0.3, 1, and 3 μg from left to right in each panel) of the Δ5 mA3-expressing plasmid added for transfection. The amount of total input DNA was kept constant between samples by the addition of the empty parental plasmid. The data represent means with standard errors from three independent experiments. *, P < 0.001; #, P < 0.01; §, P < 0.05 by one-way ANOVA with Tukey’s multiple comparison tests. (TIF) [file ppat.1008173.s002.tif]

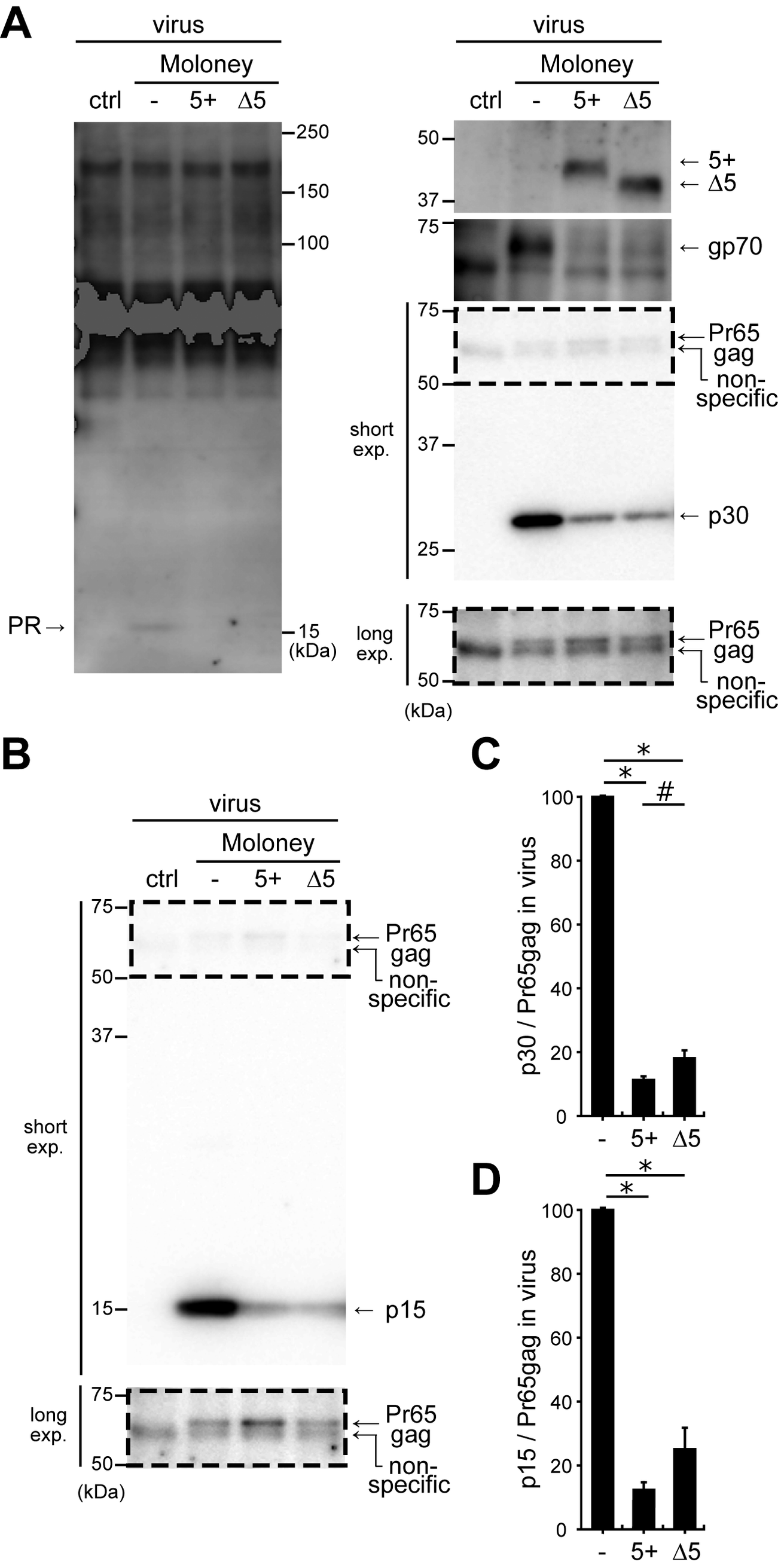

Supplement: S3 Fig — (A-D). The experiments were performed similarly to those shown in Figs 3A and 3C and S1 Fig except that Moloney MuLV was used. The goat anti-Rauscher gp70 Ab was used for the detection of M-MuLV gp70. The data represent means with standard errors from three independent experiments. *, P < 0.001; #, P < 0.05 by one-way ANOVA with Tukey’s multiple comparison tests. (TIF) [file ppat.1008173.s003.tif]

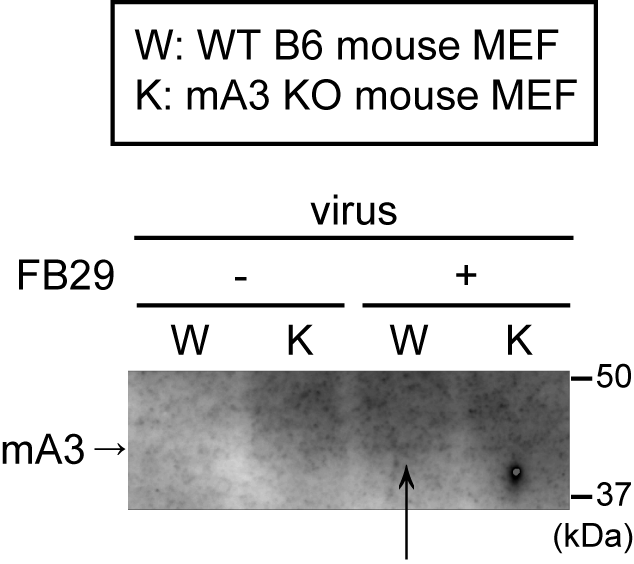

Supplement: S4 Fig — Virus lysates prepared and evaluated as shown in Fig 4A, right panel, were used to detect mA3 in FB29 virions with the pre-absorbed anti-mA3 Ab. A band of very low intensity possibly indicating the presence of WT MEF-derived mA3 was detected, but was hardly distinguishable from the background (arrow). (TIF) [file ppat.1008173.s004.tif]

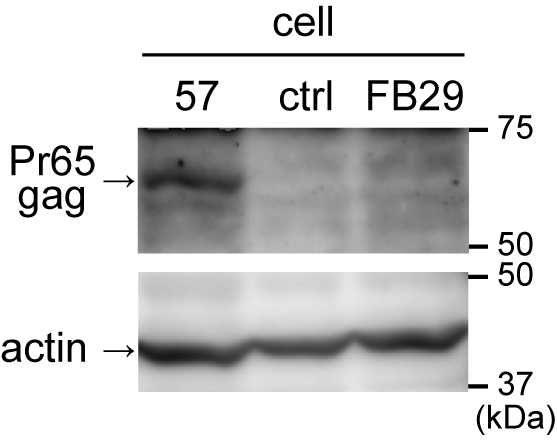

Supplement: S5 Fig — 293T cells were transfected with 6 μg of viral DNA or the control vacant plasmid (ctrl). The cells were harvested at 3 days after transfection, and analyzed by immunoblotting. Anti-p15 (MA) mAb 690 and anti-actin Ab C-11 were used to detect Pr65gag and cellular actin, respectively. (TIF) [file ppat.1008173.s005.tif]

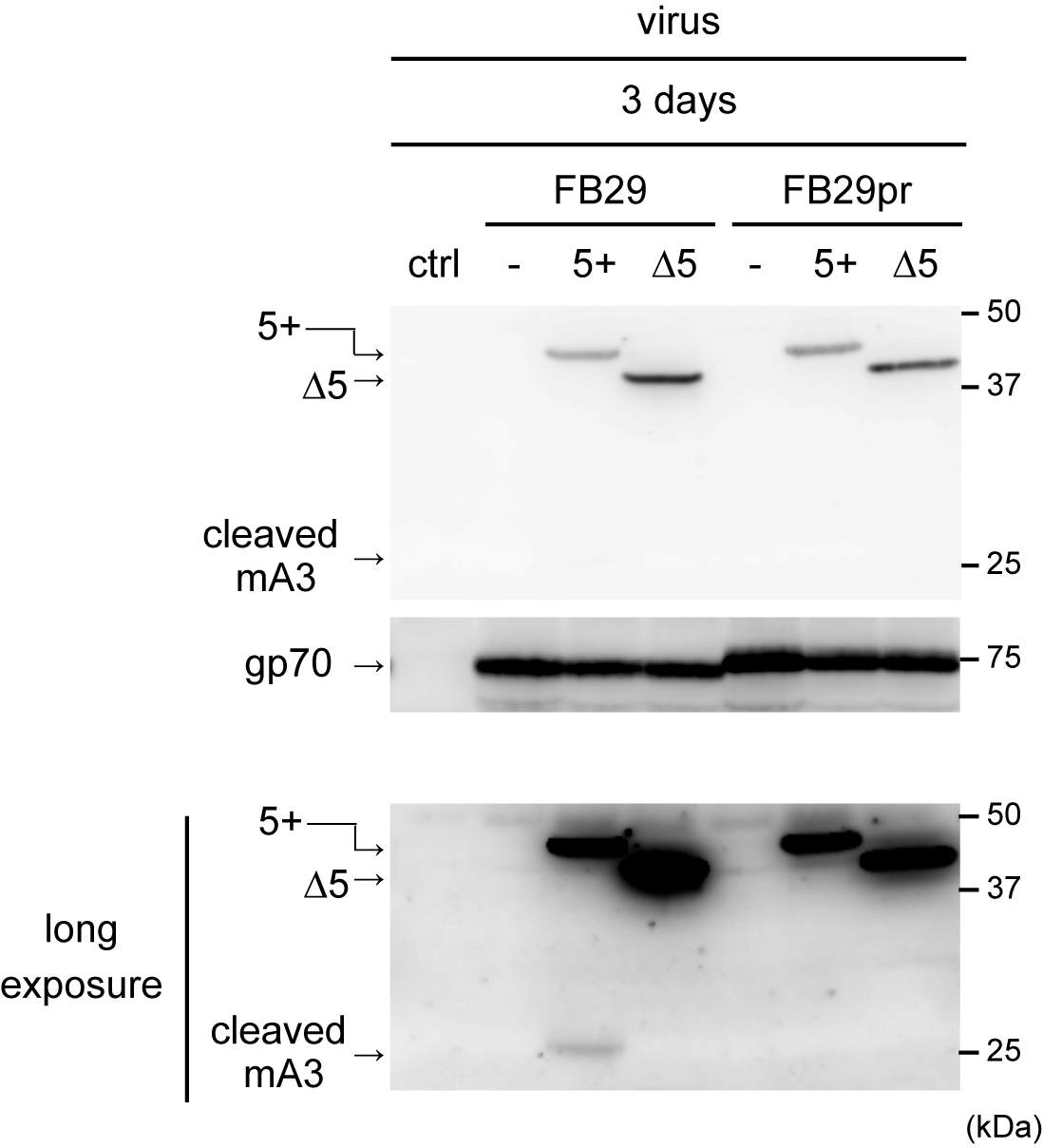

Supplement: S6 Fig — The experiment was performed similarly to that shown in Fig 2B (3 days) except by using FuGENE HD Transfection Reagent instead of Lipofectamine 3000. The virus lysates were collected at 3 days after transfection, and analyzed by immunoblotting. Anti-gp70 (SU) mAb 720 and anti-FLAG Ab M2 were used to detect gp70 and FLAG-tagged mA3 and its cleavage products, respectively. The image taken after a long exposure time for the demonstration of mA3 cleavage product is also shown in the bottom. (TIF) [file ppat.1008173.s006.tif]

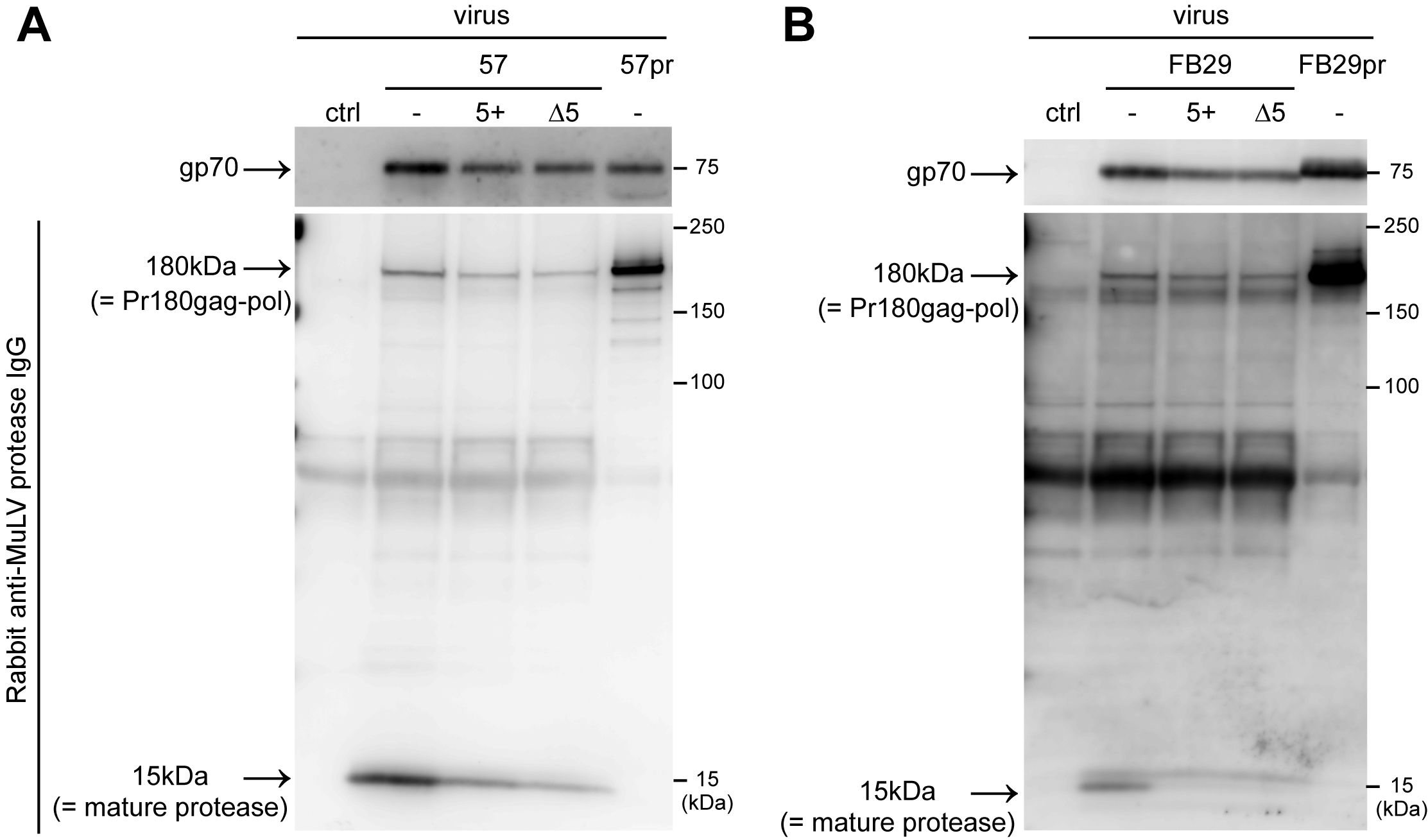

Supplement: S7 Fig — (A) The viruses were prepared as shown in Fig 2A, and analyzed by immunoblotting. Anti-gp70 (SU) mAb 720 and IgG purified from the anti-MuLV protease antiserum were used. (B) The same experiments were performed as described for panel (A) except that FB29 and the protease mutant FB29pr were used. (TIF) [file ppat.1008173.s007.tif]
